# Nuclear Pore Complex Components in the Malaria Parasite *Plasmodium berghei*

**DOI:** 10.1038/s41598-018-29590-5

**Published:** 2018-07-26

**Authors:** Jessica Kehrer, Claudia Kuss, Amparo Andres-Pons, Anna Reustle, Noa Dahan, Damien Devos, Mikhail Kudryashev, Martin Beck, Gunnar R. Mair, Friedrich Frischknecht

**Affiliations:** 1 Integrative Parasitology, Center for Infectious Diseases, Heidelberg University Medical School, Im Neuenheimer Feld 344, 69120 Heidelberg, Germany; 2 Structural and Computational Biology Unit, European Molecular Biology Laboratory, Meyerhofstrasse 1, 69117 Heidelberg, Germany; 3 Centro Andaluz de Biología del Desarrollo CABD, Universidad Pablo de Olavide-CSIC, Carretera de Utrera, 41013 Sevilla, Spain; 4 Max Planck Institute of Biophysics, Max-von-Laue Str. 3, 60438 Frankfurt am Main, Germany; 5 Buchmann Institute for Molecular Life Sciences, Goethe University of Frankfurt, Max-von-Laue Str. 17, 60438 Frankfurt am Main, Germany; 6 Iowa State University, Biomedical Sciences, College of Veterinary Medicine, 1800 Christensen Drive, Ames, IA 50011 USA

## Abstract

The nuclear pore complex (NPC) is a large macromolecular assembly of around 30 different proteins, so-called nucleoporins (Nups). Embedded in the nuclear envelope the NPC mediates bi-directional exchange between the cytoplasm and the nucleus and plays a role in transcriptional regulation that is poorly understood. NPCs display modular arrangements with an overall structure that is generally conserved among many eukaryotic phyla. However, Nups of yeast or human origin show little primary sequence conservation with those from early-branching protozoans leaving those of the malaria parasite unrecognized. Here we have combined bioinformatic and genetic methods to identify and spatially characterize Nup components in the rodent infecting parasite *Plasmodium berghei* and identified orthologs from the human malaria parasite *P. falciparum*, as well as the related apicomplexan parasite *Toxoplasma gondii*. For the first time we show the localization of selected Nups throughout the *P. berghei* life cycle. Largely restricted to apicomplexans we identify an extended C-terminal poly-proline extension in SEC13 that is essential for parasite survival and provide high-resolution images of *Plasmodium* NPCs obtained by cryo electron tomography. Our data provide the basis for full characterization of NPCs in malaria parasites, early branching unicellular eukaryotes with significant impact on human health.

## Introduction

The nuclear envelope of eukaryotes constitutes a barrier between the nucleoplasm (containing the genome) and the cytoplasm. The essential selective and bi-directional exchange of macromolecules across the nuclear envelope is mediated by the nuclear pore complex (NPC) which is assembled from around 30 different nucleoporins (Nups)^1,2^. Nups appear generally well conserved among the Opisthokonta, which include yeast, *C. elegans* and humans^3–7^. Other studies also investigated *Arabidopsis thaliana*^8^, the early branching parasite *Trypanosoma brucei*^9,10^, causative agent of sleeping sickness and very recently *Toxoplasma gondii*^11^. The NPC is a large protein assembly with a mass of approximately 60 MDa in *Saccharomyces cerevisiae* to 120 MDa in vertebrates. Nups exist in multiple copies per NPC. They establish a central pore, called the inner ring, that is sandwiched between the cytoplasmic and nuclear rings on either side. The nuclear basket and the cytoplasmic filaments comprise more peripheral structures that attach to the nuclear and cytoplasmic rings, respectively. Nups are classified into two major groups: Firstly, the intrinsically disordered Nups with frequent FG (phenylalanine-glycine) repeats that line the central channel and interact with translocating cargo complexes. Secondly scaffold Nups that make up the cylindrical architecture grouped around the central channel^1,4^. Transmembrane (TM) Nups – NDC1, GP210 and POM121 – anchor the NPC within the nuclear membrane^1^.

The major function of the NPC is the transport of molecules in and out of the nucleus. It forms a diffusion barrier^12^ with larger macromolecules interacting with nuclear transport receptors (NTRs) to form cargo complexes, which translocate through the central channel through transient interactions with FG-repeat regions^2,5^. Although the exact mechanism mediating the transport through the nuclear pore is still an active field of research, recent work indicates that the key functional features defined by biochemical properties of FG Nups and NTRs are universally conserved^13^. Additional functions of the nuclear pore complex include a role in the regulation of basic aspects of cell division, transcriptional regulation and signaling^14^. In yeast, soluble Nups can bind transcriptionally active genes and direct them to the nuclear periphery^15–18^. It is not clear whether Nups can play such a regulatory role in the malaria parasite. It is well-established that *var* genes—immunovariant surface antigens of a 60-member strong PfEMP1 protein family that undergo allelic exclusion and define antigenic variation in the human malaria parasite—are transcribed in a perinuclear localization^19–22^. The *P. falciparum* Nup116 (PF3D7_1473700) has been studied in this context but was found not to define such a peripheral compartment^23^, although other, yet unknown Nups may do so. During the intraerythrocytic developmental cycle 3–7 nuclear pores are present per nucleus in early ring stage parasites. As the parasite grows the number increases transiently up to 60 in the trophozoite before they are being distributed among the 16–32 daughter progeny^22^. The identification of NPC components and their study in the malaria parasite have been hampered by the small size of the parasite and the poor sequence homology between Nups from key eukaryotic model organisms with those from *Plasmodium*. This divergence is highlighted in a 2010 genomic study^24^. While a HMMer-based Nup screen successfully identified 19 Nups in *Trypanosoma brucei* of which 18 were confirmed experimentally^9,10,25^ the very same study lists a mere two candidates for *Plasmodium*. These candidate Nups are SEC13 and a putative homolog of Nup96–98. Both proteins had already been identified by Aravind and co-workers^26^ in 2004. These findings provide strong evidence that the NPCs characterized thus far, whether from opisthokonts, plants or trypanosomes, share a low degree of homology on the amino acid level with *Plasmodium* Nups. This suggests either an evolutionarily divergent origin of the NPC in this phylum or, perhaps more likely, loose evolutionary constraints on the primary amino acids sequence that manifest themselves in low sequence conservation. In case of FG-Nups this would be in line with the idea that biophysical properties rather than sequences are evolutionary restrained. This clearly impedes the identification of *Plasmodium* Nups.

In the human malaria parasite *P. falciparum* three proteins SEC13^27^, PF3D7_1446500^28^ and Nup116/PF3D7_1473700^23^ have been localized at the nuclear pore. A fourth protein PF3D7_0905100 with a molecular weight of 235 kDa is annotated as Nup100 in the *Plasmodium* genome repository www.plasmodb.org. Three of those proteins are conserved in the rodent malaria model parasite *P. berghei*, but Nup116 lacks a syntenic ortholog in *P. berghei*. Recently, an immunoprecipitation protocol was developed allowing the biochemical isolation of 15 NPC components from *Toxoplasma gondii* tachyzoites using TgNup302, a homolog of human Nup98–96 and yeast Nup145, as a bait^11^.

The complex *Plasmodium* life cycle encompassing both vertebrate (e.g. human or mouse) and mosquito hosts relies on the timely expression of genes restricted to specific stages within the life cycle^29^. This requires tight gene regulation and may involve components of the NPC. Encoding some 5000 genes the parasite develops a variety of life cycle forms that are at times motile, intra- or extracellular, cell cycle arrested or undergo rapid divisions. We present here the first identification of Nups in the widely used rodent malaria model parasite *P. berghei*. We provide localization data and life cycle expression analysis through tagging with the green fluorescent protein (GFP) of five Nups exploring mouse and mosquito stage parasites. Similar to the mRNA export machinery in *P. falciparum*^30^ we find that *Plasmodium* NPC components share little sequence conservation with those from other eukaryotes. The various *Plasmodium* genomes contain more than 5000 genes with more than 30% still annotated as hypothetical proteins and lacking clear homologs outside the genus.

## Results

### Bioinformatic identification of *Plasmodium berghei* Nups

Although generally conserved, *Plasmodium* Nups have been largely elusive^24,26^. In order to identify candidate proteins assembling the *Plasmodium berghei* NPC we first used BLASTP (Basic Local Alignment Search Tool) to search at *Plasmodium* gene repositories (plasmodb at www.plasmodb.org, genedb at www.genedb.org) for homologs of yeast and human Nups. The study by Courjol and Gissot with co-workers^11^ identifying the first *Toxoplasma gondii* Nups was not yet available during the initial phase of this project, and *Toxoplasma* sequences were therefore not included in the search approach outlined here. We provide a comparison of *T. gondii* with *P. berghei* Nups at all other relevant sections of this manuscript.

**Figure 1:**
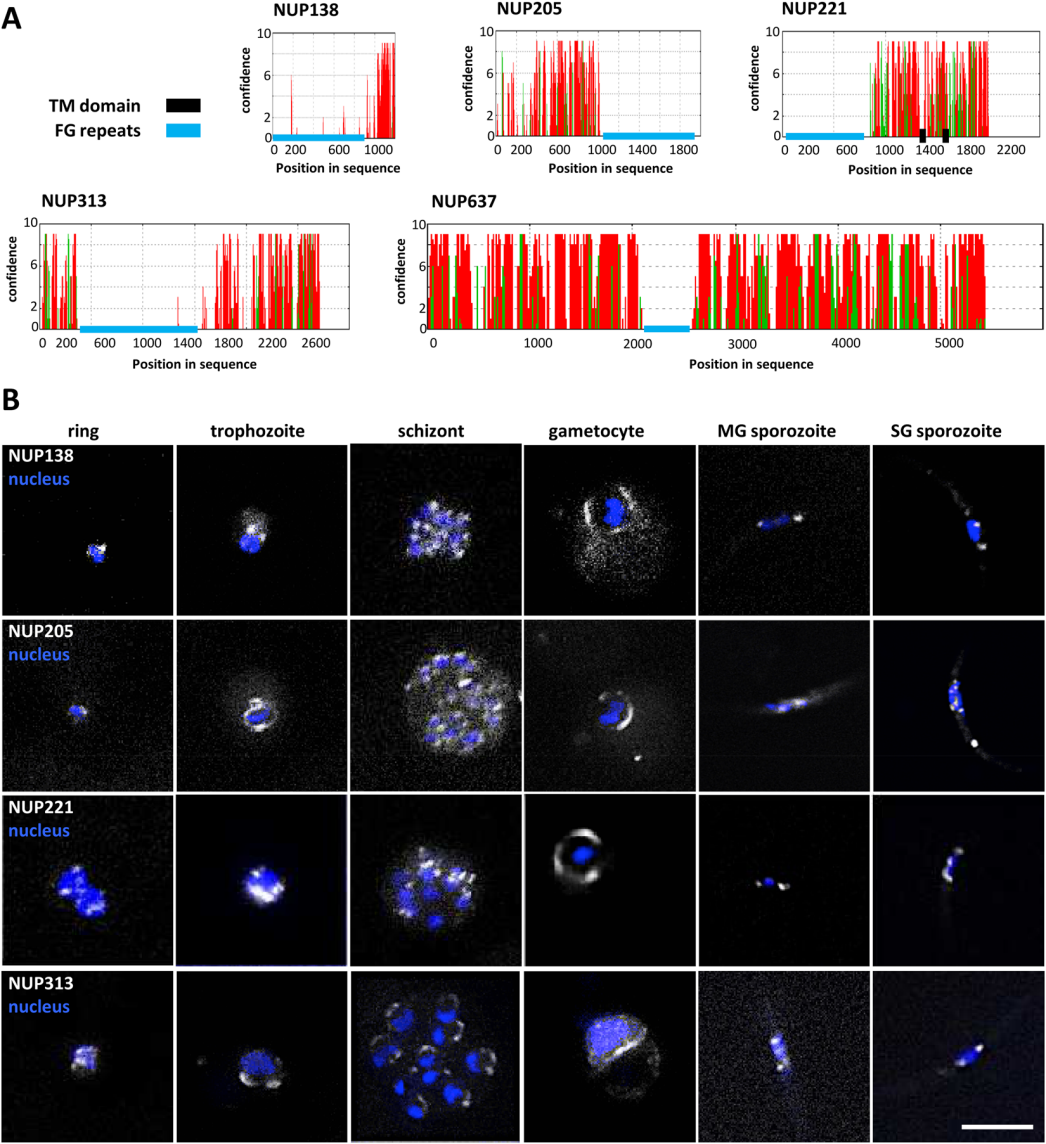
Fluorescent tagging of novel *P. berghei* FG Nups. (**A**) Fold predictions using PSIPRED of newly identified Nups. The y-axis indicates the confidence score. Red lines = alpha helices, green lines = beta sheets. Transmembrane domains and FG repeat regions are indicated by colored blocks below the the sequence. (**B**) Live imaging of Nup138, Nup221, Nup313 and Nup205 tagged with GFP (white) throughout the parasite life cycle; nuclei stained with DAPI (blue). See also Supplementary Figs 16 and 17. Scale bar: 5 µm.

BLASTP revealed only two putative *Plasmodium* Nup homologs: SEC13 (geneID PBANKA_144540) previously characterized in *P. falciparum*^27^ and PBANKA_ 0416300. The latter is annotated as Nup100/Nsp100 despite its predicted molecular weight of 221 kDa. Homologs of Nup100, such as human Nup98–96 or yeast Nup145, are encoded as larger precursor proteins that are autoproteolytically cleaved at a conserved HFS (histidine-phenylalanine-serine) site to produce two smaller molecules^31^. Notably, Toxoplasma TgNup302 retains this cleavage site^11^, while the putative *Plasmodium* Nup100 does not but contains, atypically, two predicted transmembrane domains (Nup221 in Fig. 1A).

Failing to identify further Nups by primary sequence homology to yeast or human Nups, we scanned the *P. berghei* genome for proteins containing FG di-amino-acid repeats. This screen revealed PBANKA_0416300 (Nup100) as well as PBANKA_0107600, PBANKA_0417900, PBANKA_1140100 and PBANKA_13102000 as potential FG-Nups. The latter four genes are all classified as ‘*conserved Plasmodium protein, unknown function*’ indicating the lack of homology with proteins outside the genus and a lack of conservation of domains. This includes the FG di-amino-acid repeats that are not recognized by SMART^32^, a tool allowing the identification of protein domains. The number of FG di-amino-acid repeats was smallest in PBANKA_0107600 and PBANKA_0417900 with 14; 15 were present in PBANKA_1140100; 21 in PBANKA_1310200 and 31 in PBANKA_0416300 (Supplementary Figs 1–5; these figures also provide the names of the syntenic *P. falciparum* orthologs). A total of 95 FG repeats are present in the following combinations: AXFG (n = 2), EFFG (N = 1), FXFG (n = 4), GXFG (n = 9), KGFG (n = 2), LFFG (n = 1), MFFG (n = 1), NXFG (n = 28), PKFG (n = 1), SXFG (n = 35), TXFG (n = 6), VXFG (n = 3), YLFG (n = 2). Two thirds of all FG-repeats are of the format [SN]XFG, while the commonly FXFG and GLFG variants found in yeast and humans are only present on thirteen occasions (14%).

The five *P. berghei* proteins vary in length from 1237 to 5440 amino acids with predicted molecular weights from 138 to 637 kDa. In keeping with the nomenclature in other organisms, they were assigned the following names reflecting their predicted molecular weights: Nup637 (PBANKA_0107600); Nup313 (PBANKA_1310200); Nup221 instead of Nup100 (PBANKA_0416300); Nup205 (PBANKA_1140100); and Nup138 (PBANKA_0417900). Four of the five proteins contain internal repeats (Supplementary Figs 1–5) that may have arisen through localized duplication events. One of the five FG-repeat proteins, Nup221, contains two predicted transmembrane domains (Fig. 1A; Supplementary Fig. 2) implying a potential role in NPC assembly or anchoring of the NPC in the nuclear membrane. Secondary structure predictions showed that unordered FG repeat regions are flanked by alpha helical domains that typically produce alpha-solenoid folds^10^ (Fig. 1A).

Curiously, the previously identified *P. falciparum* Nup116 (PF3D7_1473700) is not present in *P. berghei* although it is localized in a syntenic region of the genome and flanked by orthologous genes. It is the only one of the Nups presented here that is not universally conserved within the genomes of 8 different *Plasmodium* species; i.e. *P. berghei*, *P yoelii*, *P. chabaudi*, *P. falciparum*, *P. vivax*, *P. knowlesi*, *P. cynomolgi* and *P. reichenowi*.

Employing the same search strategy for the related apicomplexan parasite *Toxoplasma gondii* we scanned its genome for proteins with FG di-amino-acid repeats and identified seven putative FG Nups (Supplementary Figs 6–12): Four of those had recently been identified experimentally by Courjol and Gissot with co-workers^11^ using Tg302 as a bait during immunoprecipitation. The remaining three are TgNup145 (TGGT1_203780), TgNup206 (TGGT1_305790) and TgNup43 (TGGT1_310610). The number of FG di-amino-acid repeats ranges from fifteen to 45; the molecular weights from 43 to 593 kDa. In contrast to *P. berghei*, *T. gondii* has retained FG-Nups with clearly recognizable domains homologous to human and yeast Nups. These are *T. gondii* Nup302^11^ with an N-terminal FG repeat region separated from a Nup116_C domain by an autoproteolytic HFS signature motif as found in human Nup98-Nup96 and yeast Nup145 (Supplementary Fig. 7); or Nup69 with an NSP1_C domain (Supplementary Fig. 8) that provides interaction points with Nup57 and Nup82 in yeast^33^.

### *In vivo* localization of proteins identified by bioinformatic analyses

In order to detail the localization of the proteins obtained from the bioinformatic analyses we introduced a GFP-tag at the C-terminus of each protein. All plasmid constructs are promoter-less and require integration into the genome for protein expression of the fusion gene under the control of its own, native promoter in this haploid protozoan (Supplementary Fig. 13). Therefore, the tagged protein is the sole source of protein in each mutant. Except for Nup637, all transfections produced a transgenic parasite line. The failure to introduce the plasmid pLIS0238 into the Nup637 locus suggests a detrimental influence of the C-terminal tag on the function of this protein.

Live microscopy of transgenic lines identified GFP-fluorescence in asexual stage parasites as well as gametocytes. The staining ranged from polar and clustered, to perinuclear and contiguous (Fig. 1B), and reflects the distribution of the NPC in the different stages identified during the intraerythrocytic developmental cycle of the related human malaria parasite *P. falciparum*^22^. In ring stage *P. falciparum* parasites, the few nuclear pores (3–7) are typically clustered in a single location, a characteristic reflected in the ring stage fluorescent images. In the trophozoite the number of nuclear pores increases up to 12–58 per cell in *P. falciparum*. The fluorescence for each of the FG Nups increases with the size of the nucleus in our *P. berghei* lines. In general the associated NPC signal is more evenly distributed across the nucleus. With the development of up to 32 daughter merozoites from a single trophozoite and the concomitant decrease in pores per parasite to 2–6 as determined in *P. falciparum*^22^, the GFP signal appears again focused on one side of the nucleus. Lastly, gametocytes (sexual precursor cells) display an atypical Nup localization distant from the DNA stain. This may perhaps reflect the specific nature of these forms, which are morphologically highly distinct between *P. falciparum* (elongated) and *P. berghei* (round). When taken up during a mosquito blood meal, a single cell-cycle arrested male gametocyte will, in less than 15 minutes, produce eight individual motile gametes able to fertilize a female. In mosquito stages fluorescence was evident in oocysts, large syncytial assemblies that produce sporozoites. Sporozoites isolated from the midgut oocysts displayed a fluorescent signal closely associated with the nuclear stain. In salivary gland derived sporozoites the distribution of the signal did not alter.

### SEC13 defines the developmental progression from midgut to salivary gland sprozoites

**Figure 2:**
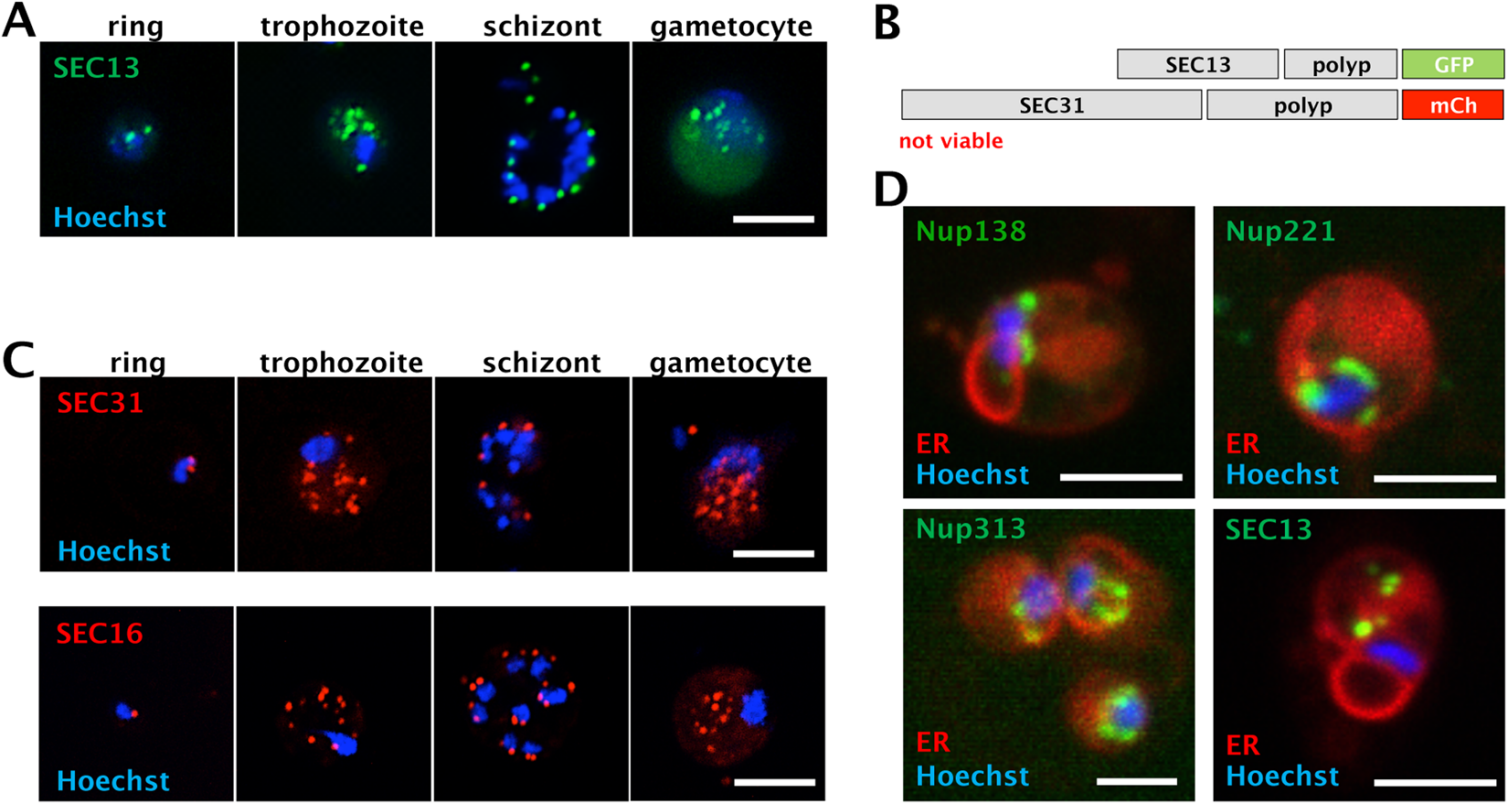
Fluorescent tagging of SEC13, SEC31 and SEC16. (**A**) SEC13-GFP localisation in blood stage parasites. Note the speckled staining removed from the nuclei. (**B**) Schematics of genetic conctructs used in the tagging of SEC13-GFP and SEC31-mCherry (mCh). A parasite line expressing both was not viable. (**C**) mCherry tagging of SEC31 and SEC16. Note the similar localisation pattern of these two COPII components with SEC13 in the different stages of the parasite. (**D**) Live imaging of GFP-tagged Nup138, Nup221, Nup313 and SEC13 with ER tracker Red in blood stage trophozoites. Scale bars: 5 µm.

While the exclusive perinuclear staining patterns of all four FG Nups was consistent with similar studies from other organisms such as *T. brucei*^10^, SEC13 (PBANKA_1445400) displayed frequent speckled localization sites away from the nucleus in several life cycle stages consistent with its inclusion in COPII vesicles (Fig. 2A). A co-localization with the COPII protein Sec31 could have differentiated between the relatively rare nuclear pores^22^ and SEC13 of the COPII vesicle coat. However, transfection of either mutant with a plasmid to introduce a tagged version of the second gene failed (Fig. 2B). This might be due to steric hindrance within the COPII vesicle coat where SEC13 and SEC31 interact intimately. Tagging with mCherry of SEC31 alone however was readily accomplished and yielded a parasite population with a signal distant from the nuclear DAPI-staining consistent with its function as a COPII vesicle protein at the tER (Fig. 2C). The distribution of staining for the COPII coat assembly protein SEC16^34^ tagged with mCherry at the C-terminus was again similar to the one for SEC31 (Fig. 2C). Co-staining with ER tracker corroborated the nuclear envelope position of FG Nups and revealed the absence of an ER-associated signal, while SEC13 was prominently localized away from the DAPI-stained nucleus (Fig. 2D). The signal was particularly strong and widespread in the trophozoite and the gametocyte (Fig. 2C).

**Figure 3:**
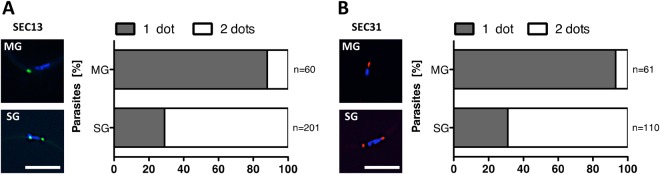
Developmental progression reflected by SEC13 and SEC31 staining patterns. Sec13-GFP (**A**) and SEC31-mCherry (**B**) localisation during midgut (MG) to salivary gland (SG) sporozoite maturation. Scale bars: 5 µm.

As for the FG Nups, we also followed SEC13 and SEC31 fluorescence throughout parasite development in the mosquito. These experiments revealed an intriguing change in fluorescence signal distribution during the maturation of midgut into salivary gland sporozoites (Fig. 3). While a single fluorescent spot is present in a juxta-nuclear position in the majority of sporozoites isolated from midguts, a second, equally strong signal appeared on the opposite side of the nucleus in sporozoites isolated from salivary glands. Sporozoites are latent, cell cycle-arrested forms that are invasive for salivary glands (if derived from midguts), where they mature and become highly invasive for liver cells^35^. The staining of these two proteins was different in respect to the FG Nup staining as all FG Nups showed more than just one localization spot in midgut derived sporozoites (Fig. 1B). This indicates that SEC13 and SEC31 are not associated with (all) NPCs at the sporozoite stage. While the appearance of a second SEC13 and SEC31 spot correlates with sporozoite progression and hence maturation we can currently only speculate about its functional relevance.

### A unique C-terminal SEC13 extension is required for life cycle progression

**Figure 4:**
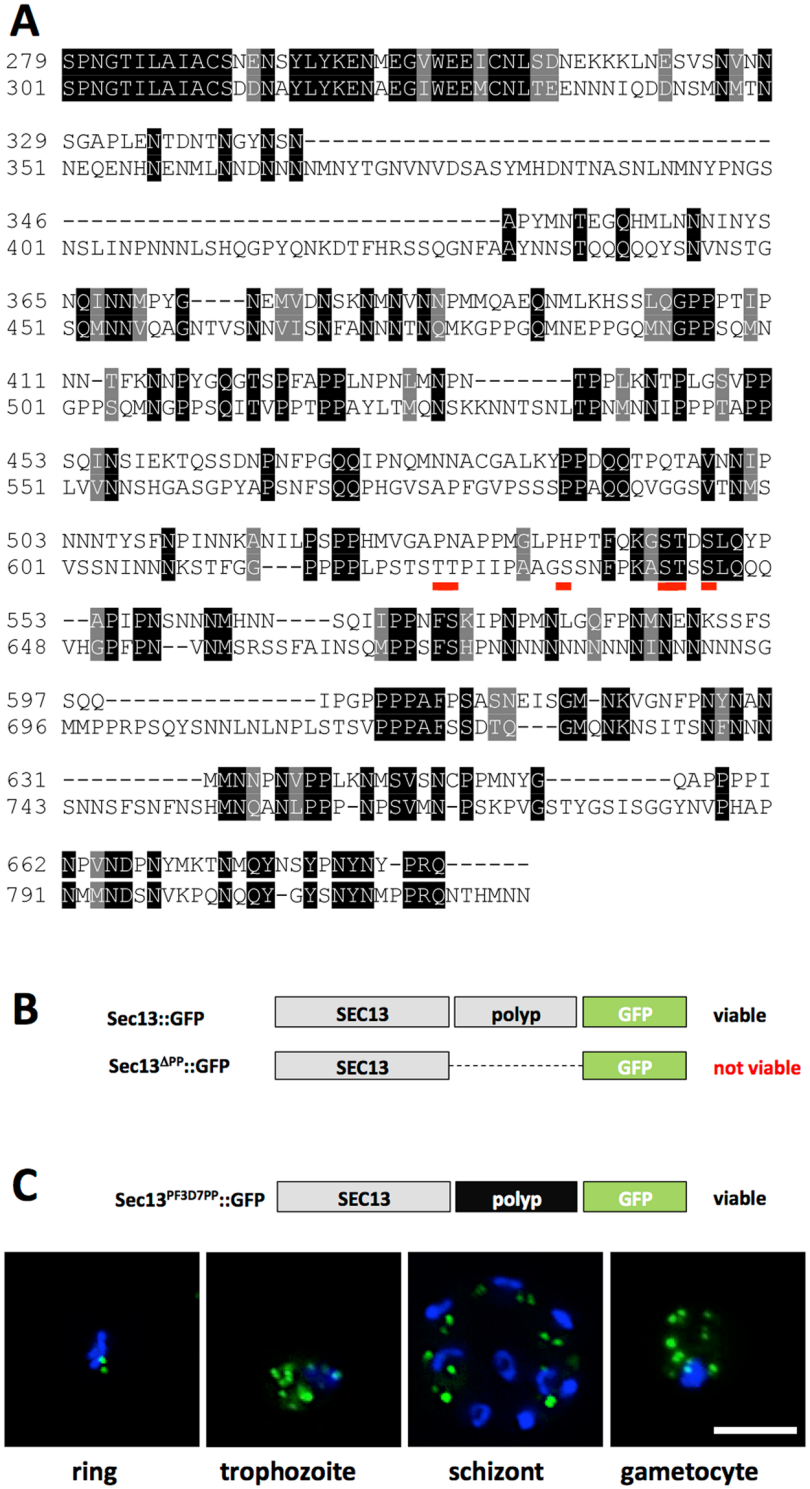
Alignment of the C-termini of SEC13 from rodent (*P. berghei*) and human (*P. falciparum*) infecting malaria species, and polyproline exchange strategy. (**A**) ClustalW aligenment of the SEC13 C-terminal polyproline domain from *P. berghei* (top sequence) and *P. falciparum* (bottom sequence) starting with the highly conserved, sixth WD-domain of both proteins. Red underlined are six documented phosphorylation sites in the *P. falciparum* protein. (**B**) Schematic summary of viable and non-viable *P. berghei* mutants. (**C**) Live imaging of SEC13^PF3D7PP^ shows unaltered protein localisation. Scale bar: 5 µm.

In *P. berghei* the ortholog of SEC13 contains a large C-terminal extension enriched in proline residues (Fig. 4A). This unordered domain is reminiscent of the C-terminal part in *P. berghei* SEC31, its binding partner in COPII vesicles. SEC31 also shows a similar, loosely conserved polyproline-rich stretch in *P. falciparum* (Supplementary Fig. 14) and *S. cerevisiae*. The large, C-terminal extension of SEC13 is conserved in many *Plasmodium* species and is present in the related apicomplexans *Cryptosporidium parvum* and *C. muris* (but not *C. hominis*, perhaps due to a sequencing error; the repetitive CQQLL may be an indication to that), *Toxoplasma gondii* (Supplementary Fig. 15) and *Neospora caninum*. It is also found in the unicellular haploid red alga *Cyanidioschyzon merolae*. These proteins are listed under orthomcl group OG5_127687. The number of proline residues in these SEC13 proteins ranges from 73 to 115. While individual proline residues within the SEC13 polyproline stretch of *P. berghei* are well conserved in the closely related *P. yoelii* parasite, another rodent malaria species, the amino acid sequence in *P. falciparum* is highly divergent and aligns poorly (Fig. 4A). In order to address a possible function for this C-terminal extension we attempted the generation of a *P. berghei* mutant lacking this C-terminal polyproline motif (Fig. 4B). This was not possible indicating that this domain provides an essential role for this protein for parasite survival, either as part of COPII vesicles, or as a Nup, perhaps as an unusual SEC13-Nup145C fusion as suggested for *P. falciparum*^27^. Despite the large sequence divergence between *P. berghei* and *P. falciparum* (Fig. 4A) we were readily able to produce a mutant parasite line that expressed the polyproline motif of *P. falciparum* SEC13 in place of the *P. berghei* SEC13 polyproline motif (Fig. 4C). The staining pattern of this protein matched that of wildtype-like *P. berghei* SEC13-GFP.

### Cryo-electron tomography confirms a regular morphology of the *P. berghei* NPC

**Figure 5:**
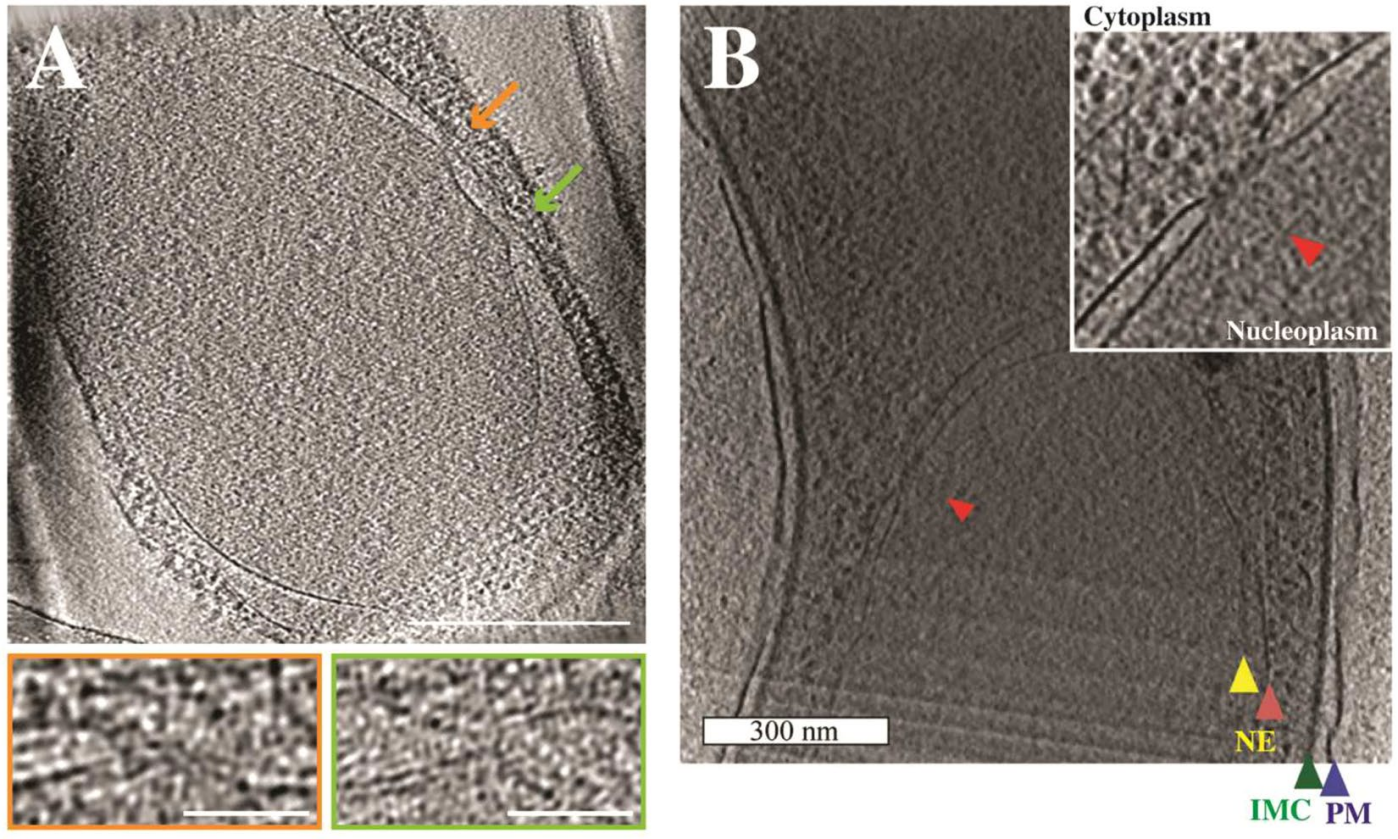
Cryogenic electron tomography of nuclear pore complexes. (**A**) Slice through a tomogram showing the nucleus of a midgut sporozoite with arrows indicating the location of two nuclear pore complexes. Enlarged images in the coloured outtakes indicate the flattening of the nuclear membrane at the NPCs. Scale bars: 300 nm (large image), 50 nm (small images). (**B**) Part of a midgut sporozoite nucleus with one nuclear pore indicated with a red arrowhead. The other arrowheads indicate the inner (yellow) and outer (pale red) leaflet of the nuclear envelope, the inner membrane complex (green) and the plasma membrane (blue) of the sporozoite. Scale bar: 300 nm. The inset shows the NPC in stronger contrast.

In order to provide a better structural display of the *Plasmodium* nuclear pore we chose the thinnest parasite form, the extracellular sporozoite for analysis by cryo-electron tomography. The nucleus is located at the thickest region of the sporozoite, which nevertheless made it challenging to record tomograms^36^ without sectioning or FIB-milling. However, we recorded over 10 tomograms of the nuclear area from sporozoites isolated from the mosquito midgut (Fig. 5), which are generally thinner than sporozoites isolated from the salivary gland. We noted NPCs in the nuclear envelope and estimated from our tomograms that there are likely not more than 10 NPCs per sporozoite nucleus^36^. This estimate is based on the low numbers (usually 1–2 per tomogram) of NPCs found for sporozoites isolated from the midgut. Although most tomograms contained the entire nucleus the missing wedge prevented a complete identification of all NPCs. Similarly, the extracellular and invasive *T. gondii* tachyzoites were found to contain 4–8 nuclear pores^11^ and thus in the same range as reported for *P. falciparum*^22^. The *Plasmodium* NPC observed by cryo-electron tomography appears morphologically similar to other species in terms of the size of the central channel and membrane distance. A more detailed examination, however, would necessitate the imaging of hundreds of sporozoites to yield high enough NPC numbers of subtomogram averaging. The quality and frequency of images we obtained did not encourage us to do so.

## Discussion

The nuclear pore complex plays a key role in chromatin organization. Also some NPC proteins can locate transcriptionally active genes to the nuclear periphery. So far, little is known about the involvement of NPCs in gene regulation in *Plasmodium* parasites. Furthermore, the closed mitosis employed by this parasite will likely require access of spindle regulating factors to the nucleus. This could necessitate cell cycle specific alterations of the NPC, as documented in yeast^37^. However, such studies are hampered by the ill-defined nature of the NPC in *Plasmodium*. The results shown here provide first steps into identifying the composition of the nuclear pore complex in the rodent model malaria parasite *P. berghei*. We have identified SEC13 and five new FG Nups and imaged four of these (Nup138, Nup221, Nup313 and Nup205) by GFP-tagging across the life cycle providing first views of the NPC in the sporozoite, the stage infectious to the mammalian host.

The five FG Nups contain a total of 95 FG repeats, between fourteen and 31 FG per protein. The amino acid composition of these repeats is evolutionarily unique; a mere five adhere to the signature GFLG tetrad found in the majority of organisms, while 63 out of 95 are [SN]XFG. In addition, the *P. berghei* FG Nup221 contains two putative transmembrane domains that could help embed the *Plasmodium* NPC into the nuclear membrane. The NPC anchor is traditionally provided by three transmembrane Nups: nuclear division cycle (NDC) 1, glycoprotein (GP) 210 and pore membrane protein (POM) 121 in humans. In yeast NDC1, POM33, and POM152 perform this function^1^. However, orthologs to these could not be detected in the available *Plasmodium* genomes.

As an extension to work on *P. falciparum* Sec13^27^, we explored here the localization of *P. berghei* SEC13 and the role for its unique C-terminal extension. The specific role of this proline-rich domain is unknown, but our failure to generate mutants lacking this domain suggests that it is essential. The domain is present in the genus *Plasmodium* as well as in *Toxoplasma gondii* and a number of related species. The fact that SEC13 does localize at the apical or proximal end of nuclei in sporozoites is curious as none of the other NUPs shows this exclusive staining (Figs 1B and 3). This suggests that SEC13 might not localize to NPCs or only to a subset. Furthermore, the appearance of a second spot in salivary gland derived sporozoites hints at a specific function, possibly in secretion, as these forms need to migrate rapidly, which necessitate the secretion of different proteins^35,38,39^. However, in the absence of a viable mutant this remains speculation.

With the exception of SEC13, which is also a member of the COPII vesicular trafficking complex, all Nups localized exclusively to the nuclear periphery in different stages of the parasite life cycle. We could also show that the number and distribution of these nucleoporins varies across the life cycle. This might reflect constraints imposed by transcriptional activity in the parasite, specifically highlighting differences in forms that are cell cycle arrested and those undergoing DNA replication. If these NPC play important roles in gene regulation and life cycle regulation they might even constitute suitable drug targets for anti-malaria drugs owing to their low sequence homologies with human Nups.

Despite current and past work, the composition of the *Plasmodium* NPC is still largely unknown^21,22,27,40^. The typical NPC contains around 30 different proteins^1^. An immunoprecipitation approach using SEC13 with an aim to identify subunits of a potential Nup84 subcomplex has been performed in *P. falciparum*^27^. The results corroborated the inclusion of the protein in COPII vesicles identifying in the eluates SEC31, SEC. 23, SEC. 24b and a beta subunit of the coatamer complex likely related to the vacuolar-associated SEA complex. While the identification of eight RNA/DNA binding proteins and one nuclear transport protein confirm that SEC13 plays a role in trafficking at or through the NPC, the study failed to identify NPC components^27^. In contrast to *P. falciparum*, immunoprecipitation with SEC13 in *T. brucei* produced Nup158 (with FG repeats), Nup152, Nup132, Nup89, Nup82 and Nup41; all components of the outer NPC ring^10^. The identification of novel Nups in *Plasmodium* may aid the biochemical identification of the NPC composition providing multiple handles that can now be explored with immunoprecipitation assays. This could be accomplished following the approach used by Dahan-Pasternak and Dzikowksi^27^ or Courjol and Gissot with co-workers^11^, or by proximity-based biotinylation methods established for osmiophilic bodies, small secretory vesicles in sexual stage malaria parasites^41,42^. For the latter it is important to note that we could C-terminally tag several Nups with GFP, hence tagging with BirA or APEX for bioID should also yield viable parasite lines. These approaches should disclose new NPC components and reveal further insights into NPC architecture and distribution in apicomplexan parasites, as well as their evolutionary origin outside the Opisthokonta. The *Plasmodium* NPC composition is distinct with little conservation to nucleoporins identified in other organisms. This notion is best reflected in the lack of conservation with recently identified *Toxoplasma* Nups^11^, and the unique C-terminal extension of SEC13. This proline-rich domain is also found in the COPII coat protein SEC31 reflecting perhaps the common ancestry of vesicle coat complexes and the NPC^43^. With little sequence conservation evident it is possible that unique features of the Plasmodium NPC will be identified, which will be of fundamental interest for the understanding of nuclear pore complex function and evolution.

## Materials and Methods

### FG di-amino-acid repeat identification

The entire protein coding genome was downloaded from plasmodb (release 28) and scanned for FG dinucleotide repeats. We identified PBANKA_0107600 (14 FG repeats), PBANKA_0416300 (annotated as nucleoporin Nup100) (31 FG), PBANKA_0417900 (14 FG), PBANKA_1140100 (15 FG), and PBANKA_1310200 (21 FG).

### Fluorescent protein tagging

C-terminal regions of all FG Nups, SEC13, SEC31 and SEC16 were PCR-amplified from wild type genomic DNA using Phusion or HiFi Taq polymerase. Amplicons were cloned into plasmid pLIS0010 to yield an in-frame, C-terminal fusion with GFP or mCherry^41,42^. Each plasmid contains the human DHFR or *Toxoplasma gondii* DHFR selection marker for pyrimethamine-mediated selection of transfected parasites and schizonts were generated following established protocols^44^ and transfected using AMAXA technology; from day 2 onward mutant parasites were selected with pyrimethamine supplied in drinking water (*ad libitum*) until parasitemias had reached 2%. Transfectants were genotyped by PCR as indicated in Supplementary Fig. 13; clonal lines were established using limiting dilution. All gene technology experiments were performed according to national guidelines and all gene technology work is registered and was approved by the Regierungspräsidium in Tübingen.

### Parasite maintenance – mice and mosquitos

*Plasmodium berghei* strain ANKA and all lines presented in this paper were maintained in NMRI mice through serial blood passage. Mosquito stage parasites were obtained in *Anopheles stephensi*; mice infected with *P. berghei* (wild type or any of the mutants) were anaesthetised and 50 mosquitoes allowed to feed for 20 minutes. The presence of midgut oocysts was routinely checked on day 10 post infection; salivary gland sporozoites were obtained between day 17 and 22 post infection. Animal experiments were conducted according to the German guidelines and regulations and were approved beforehand by the Regierungspräsidium Karlsruhe.

### Live microscopy of *Plasmodium berghei*

Nup-GFP expression across the life cycle was performed for blood stage parasites in the presence of Hoechst-33342; oocysts, oocyst-derived midgut sporozoites and salivary gland sporozoites were isolated from infected *A. stephensi* and mounted in Hoechst-33342/PBS. For staining of the ER, parasites were incubated at 37 °C for 30 min in HBSS/Mg/Ca containing ER-Tracker Red (1 µM Molecular Probes). Cells were washed and imaged in RPMI medium containing Hoechst 1 mg/ml. Imaging of parasites was either performed on a Zeiss 200 M Axiovert widefield microscope (63x, N.A. 1.4) or on a Nikon Ti Spinning disc microscope (100x, N.A. 1.4). Images were captured with the minimal necessary exposure time (usually around 100 ms) and processed with ImageJ for optimal contrast. First, 16 bit images of all channels obtained at the microscope were converted into 8 bit before adjusting the fluorescent signal using the brightness and contrast function. Single channels were merged with the colors of choice and the cell of interest cropped to a size of 160 × 160 pixels. For each fluorescent line dozens of images were taken and representative examples are shown in the figures.

### Cryo electron tomography

*Plasmodium berghei* sporozoites were isolated from the midgut of infected *Anopheles stephensi* mosquitoes by dissection and placed in RPMI media supplemented with BAS onto holey EM carbon grids, incubated for 10–20 minutes and rapidly frozen after removal of excess liquid. The frozen grids were mounted in a cryo-holder and imaged by electron microscopy as described previously^45,46^. We focused on imaging nuclei from sporozoites, which are located centrally within the sporozoites with a shift towards the rear end of the cell. Tilt series of low-dose images were recorded using a Phillips CM 300 at a magnification of 51.000 and an objective lens defocus of −5 to −15 μm or a FEI Polara at a magnification of 18.000 and a defocus of −10 μm; both operated at 300 kV and equipped with a field emission gun and a Gatan post column energy filter (GIF 2002) with 2k × 2k pixel CCD camera (Gatan). Tomographic reconstructions were calculated by weighted back-projection using the EM-image processing package^47^ or IMOD^48^. In total 10 tomographic reconstructions of the nuclei were performed.

## Electronic supplementary material

Supplementary figures
