# Supplementary material for: Nuclear Pore Complex Components in the Malaria Parasite Plasmodium berghei
Source: Sci Rep. 2018 Jul 26;8:11249. doi: 10.1038/s41598-018-29590-5 (PMC6062611; doi:10.1038/s41598-018-29590-5)
Supplement: Supplementary file 1 — Supplementary figures [file 41598_2018_29590_MOESM1_ESM.pdf]

Supplementary material for

Nuclear Pore Complex Components in the Malaria Parasite *Plasmodium berghei*

Jessica Kehrer, Claudia Kuss, Amparo Andres-Pons, Anna Reustle, Noa Dahan-Pasternak, Damien Devos, Mikhail Kudryashev, Martin Beck, Gunnar R. Mair and Friedrich Frischknecht

Contents:
-Supplementary Fig. S1
-Supplementary Fig. S2
-Supplementary Fig. S3
-Supplementary Fig. S4
-Supplementary Fig. S5
-Supplementary Fig. S6
-Supplementary Fig. S7
-Supplementary Fig. S8
-Supplementary Fig. S9
-Supplementary Fig. S10
-Supplementary Fig. S11
-Supplementary Fig. S12
-Supplementary Fig. S13
-Supplementary Fig. S14
-Supplementary Fig. S15
-Supplementary Fig. S16
-Supplementary Fig. S17

Plasmodium berghei **NUP637**
P. falciparum **syntenic ortholog PF3D7_0609000**

>PBANKA_0107600 | Plasmodium berghei ANKA | conserved Plasmodium protein, unknown function | protein | length=5440

```
MNNLIKTMNISEGNDKLVGGGKVDESIRKENNKKQKKEHNTSFYYEVYHKLKIYYEYSIN
EEERKNKCFLKNKNPLTEITKLQDELVSFFDGNIELFSNPFYFLNYEEDNNINKNDLKNY
LGTKYPNLLNIMEDVIKCSLIINRNVYYVCDLFNDVYKNIEKNNIHTEISKTIYINEVLK
ECIEKENLIIMIFLEILIYINRNEKIGITLDNDNKSMKHEYFSEKEDNYTDNELKKNEEV
KSSSDNSSLTNNSLASLNISKKKKKKNINNKKINGDKFSYYVLVNLTENDFVENIFLEID
KIIKFLIKFDGICDKAEYAFNYKTHKINILFNLLDLLFLYFSKFHANINNISSIFKSINH
LVSTKYFDFISTDYYVKELDNLLFLESQKKNKKIIKYIKNIKNSKLISQVDDYYKLDINI
SDSGLNKFDIFSENGLNSSVYKSLNFEPSTIFPKINQSLESGITNFQGKNVSNPISIGNT
NSIPNIIITEPNENMSNINQKTQNLVDVDTLNKFSNNIVTNNQINYSNFHSEINNLRGKN
SGNTMINPQNKCGIKNNIFNKCLDCTGYWYSCNCSLNNFEKSTLTLSTQISLLLILCLHP
NIDKYVYEKRKNIKINNSSSPDGINISVQSSGKENINELLELKKKKIDFENFQTPTIWKY
NCMSTEKDYKHLMGIKNNYIISENYISENISYYYLFFSKITEAKKKKKYNKIEEKPVEEF
WNDKIFQDASNDGITGSCSYDSNILMLKFVIGLFINSKEDILSSIFDDNIFKVLRNFILK
KISSYTLIGNLIFHLFHSIFLSAFIKNGRTTDVWNTFIDSHIKRDFNLKRKKKIDNKDAN
IFKDTITSSNNNTASFIQDNAQTRQNFDGINKNINISQHRMGSDSLLNRTIQNPSDLYMH
QNSYRNMHNNIIYIYKKKGEYIIDILAFLKILCINYPRLITKYVCILKNIINRYHSRIME
FSEIDDTFYHHNLEQIHEIDNSGSRNGVNTIPTRNSKNEHDKYSQLKENKYYNINEERRM
IIMKYRSLKLIAEEKMKICIDFYSDIFVQILDFASVLCNNIYDLKIIENVVMCFKTPLTA
NLNIFNLIKKLFNQFTCALNGKFNEFSKITNKQHIYEKTRGNTKSGVVLNGNFNIIDDET
SNDITNNGFIDSINIDSTNNGQVNVKNSRDLYRNVFGRSVNISSNQNATTSSNNNVSLFS
SYKDGNGKNGNGEEEYENGNYNNSYDGNDYRYDDNDDDYCSNAYFTYINYFKLNLLEKLF
EQNNYLKEILKNNNVMEYLEKNNAYLRNLIKENYLVEKHINDKKIFKNYASNLDAYNKLL
SYENDYTASARDANKILQDIEQSNNKLKLFLNFKGVTSLFGAQVNPISYVGGLQNNLDNN
SLQLQSSMSNFLENLLEEKNCINYLLADNKVAENLLRENNILKYEYNKYNMIDLFFKNKT
EENQLDNIKSIMLTEYNVNKIFSYEKRFKILKTNFDGSYNNKEAIKFFFEDNKEAINFFK
DKKNEPPIFKDDDSSSLNENIMNRAKVILKRELEDDSKSYIDARYLLNNIFEKRFYPYNC
IKMLEKCLLFLSSVNNNKLCYYMPLNKNLLVDYILLEQYENASGNTASSTSMDIANMHLQ
ENNNVNKLFPDDDEKNPFRIRNKKYNNMISLIDTQNYINTTQFKNVFIHNLNMHSNKKSK
DKHKIVKHYKTLHLLDMLYEIVKTKGNSSLDLLNLKCLSLEILCSSFVKNSSSALSVLCI
ITNIFPDLFLDVVKQFKDQNDQLKKKLDFLDKEASIENPKYKKIEQLTNNLSVPKINLKV
IIIFMKCVNFLIYIIGIKRKFNMSLTKIWHFFNNIEKIKEIDAKMKSKKPMNSNSSTKTQ
NCAFFDIFYNFDDFFDEIDNEQNSEDAENCQNRNNNGQEFNILNMGKSNNIGNKICIESD
QEKNGKNIKNNLEQGMNNNSTIGNNIKEINSNDFEDNENNDIYEKCLKKKLMISDDDKNV
CKHLIVLTNYIVQNIFYNLINNFILKKIRDQKYEAEDFLDENIKLEIFTNKQENSMFKRR
YNYYINKSKNIESQIITMQNDVNINTSNIYQIEQMLRQNNITFEPYINSQKMFDKAFSSN
LGGNLSASIPTNSLNPFPTNFGIGTSTSQLGTSPIALGNSTSMSLFKQDNKGTNFSSMFN
SSNTQTNILSGTSGNTLNNTSSLSTSNFNNSTNFLNPSQTNTTSHSIMDSQRTMFASTSR
LLSPSQETLGLNSSSNNNTIRDASNSSLFNAPISSNNNIFKPQTGLNMFSTNSGTNTSSI
FSTPQSNTTGLFGGSSLGNNNSNINDSRSSSIFGSNMQTNKNNPTISSFGSMLNQTSSTS
NTGTNSLGLSGGFGLGSRPGMNTSNNLLLNNMDSKNSQLGGTLGASTSTSLFGANRSNST
LTNNTIFSSTTNTNSLLNPISQNNSTSNIFNKNPLGTSNTISNNSNLFSSPTASTSLFGA
NTTSQISSFGNTTSNMTQTTTSSLFNNSFGSQQNNITSSNNLLGLSSNNLALTGQGMNQQ
SSLFSNNSTGFSNNLMGNKGNSSYLFGNETNQNVMNNNASLFSTGNIYSHLGNEHNLQNI
LNREGINNINKLKEYYEDLKIKTNVLNAEIYKNKMELKKNEYNLQKEKRIQLIKEAKLRY
MKSKSDINFKEKEMYILIVLVFMYFKLILKGPLPSTKNNKKLNTVMNERFDYLCDFRPTM
YLFEQILGESNRFMNTFFNIIFLDNILHEENKNLEKIISKYKNDSDYITIYNKKKTLFRT
NIEEEDDENKISDQFKDFKNIKNNLFTTLTPETYYKKKMEYIICSYSKESYNNILFHKIK
SLGLSILKILFERDVLFIHMYNQWKNEKILYEKINKFHLNFNSINNNETSMNNMMNLNVN
DSISESILNNNNNNIITSNFNDINSRGGEQIKEGLEAGASTGGDGKEINFTESLCTPICV
HNFLFKNININSSTTYLILLLKNFFRTSEINKIIIYFILQIIIRDTKTTINILKRDAESF
NYLKYALKNIFIYNLNQQKFNNSYIISNREIKMQNILNNFIDIPLLYYLKKKNNIKTKDS
VISTRKDREFYERNKKNSISGNEVLNTSNWLSYINKHNSGNNEMDEIDLIYRVEKNKLES
ENSNMVLKTKKDGNYLFEEIEEGNSANSSYSTYNSYSSDISDNEYKKINNLKNKGHIGLN
DNYSLRNEMILKYKNFKLKNSKRNKSKYVYDYLSDIDLIDTIKSKKICIFYDNKEKDEEN
DNITFYNLSSENINDNYSKNDYYNEIKSINYNVTGFCSSYNFLPINQFYTEKYDFIESEL
VYISDLKLYFYIKSFKRHSYFTKLLNIEKVSATSENDKYKTNTSENINDDINYEIKENNL
SFSNLRRSGFDDSSLTSNKKIIINENDIIKYQNCEEKDIKFRTNNKLESENEQNDVNKIV
EDEIKVFDFYNKLNKLNENMLFPNIFYDNKVDKMKYIEYIKKIAEKEQKENDGKYKNILN
PINNILKTQKNEIEKQLSIELQKKNIYINNEYCEWIELSHLLNVDIDYNEIRGGLNKNNS
VLNNYSRMPITKLILYFYEVITRKFLFLNNSDSLIESCVLSLLGLSFTPSKQMDKKNIIT
NIDESLELEENCFLNSLIKNVIVNFSNYEHVSETIKVGYNNNIAEIGEQHNDRKKRLLTN
EKLNEFEFKDDLFINGQDEEEIILGNYINKADNENNFLNNENLYEMGSGLNNDNSKIVFL
NKKRKDYYKKNIFLNNSNNLKQYNTFEKNIYFIKSLNIIYMLSKNKKIKEYIINLINTMW
HNKYNVFYYITQNVNIEKLKDTEKIIFFKISLHILNIFIPILDHILINIDKHVEKFINLS
FPNDKEDNNSIQIFESINNNATNNELGNKNNNIGPQKYCNIFHIINKENFNEKKNLFEFL
NPIFTFDNAQNFINHYINIVKNYNDNYLKHYIYHYYLFNYNFEFQKIYNYSNLEHIHYIK
SNKLCNDINEKCFNTKSRINEYNKKNVHLLYTNIINVYVNFCKILNSYKVSKHVNVNYTF
NDGKYVENYENNNIDENVKNKILKPINIENENEFNFENLNEIIGNKSYLLKFLDDYYINE
NISKEEKKEHIEKNVYIYNSINYLNINYKNWLLVYKTYIEKITYLIDLILKIKNNTIKKK
IYYLKNYFYIVNNIQRHLKSVTSLIKSSYYINFNIQITPILFIVYLFITIFFLSHNNKFA
FKYLSCINKGFHNLFKGKNMLDFKNTENIDGEDDTNNDDDSGSEILKHNNKKNEINKKIK
```

```
FYDEINGSKQGKESDAHLCDNDEFKIDHLKYNKKLKMSSVFSKQILTKEFQNNIINIPKA
YNQDGSSNIINNFDKKTRTRYNNTSSSIHDDGIDTISNLQKGELYKSVFFINNNYNNNNN
NSNNVGSKNLDDNKLYYDIHSEYYKLILKSNEFFGDLLQLLIILITKKHPVLNKYTIIYI
YECLYTLLNIYHYSYCFAEKEYNSKNNKNMFISLIKRIDHNILNEFISTLFTDSYKNFFT
NNNSTIPIYFYNNIYKFIDYNTEIFMIKNREELTDNASILESKNRVSMHNLIDSDNSDIG
NYFLNKEQMYMKEKKKVHFADSEPTKIVNENKVDVKMSEEDNGVINLNFKNNFENKIHLN
SRSLNLYEEIYCSNFLYNKNDLNLLSINLLIFLLTRIGIYEIDIKIDENNVKNLIKGNLF
IFNKNSGNENENKILHTCFLLSALCDTSYINEFISSEDLFSLFLKSNIFNNLYEYKFNYL
SNSISFLTFPTNFLENKNIYIPLPMLIISYITVILKVIDKKGLQLFNIIGKWITTNITIF
TNHLIVNNYLMKSYEIVHEINNFTTYNHNLVYKCLCYASTCNFTREPSNNTSNSSNKPLN
FVHESKTDVKTNKENDINDNTKYTENLINHSNVKLVNLDIIYSSTYYLLRLYKNYLLYIH
QNYLNLKKLKIVENINKKKNNKSKKIKQNLTYMEKKLINKKGNIGEIDSSKNINRYINKF
NDENNIPNKIPIQKENYFRNHFHPNYSEYESNEHNSDYDESESQDNEEEEEEEDEEEEEE
KNIREMDSIKNNFSKLINSVYNEKNSQFIIGNYNNKNVLSNNMNTIFGNSNSNNGFIENN
YFYIDKGLCFEFNEISLNICNIIKEHTYILQYVKDLLNILSIFVIYDISWKDENELVTTD
IDSHKETRGNHYVNVKSDNNIDENNYNFDTKEMRRKNIRTHRILQNIIPKYSIKFHCINL
CLDIIEYDIGIYDEYVQQYKTKYYEYIKIVLKVFMNTSFYYINIIKYENKHMLKSVSVAM
KKLTNIIFFLLTNLIRKEKTTDEDKKKHALINKFLYDKNGFKKNAHEITTELFNCEDNIF
TDKKCSLDHVKKFLENTEIDIHVLKKILTCIIYFTFAMLNNQKVNKINIENINLNHINKI
NLKKIANTYLENNNILKVFTFILQRSTQIYYLLYNYAMQS
```

Figure S1. FG repeat *Plasmodium berghei* protein (sequence from release 28 of plasmodb.org). FG di-amino-acids are highlighted in blue.

Plasmodium berghei **NUP221**

P. falciparum **syntenic ortholog PF3D7_0905100**

>PBANKA_0416300 | Plasmodium berghei ANKA | nucleoporin NUP100/NSP100, putative (NUP100) | protein | length=2004

MYMQNSSSNNVFGSYNNQGNNLNKGLFGNAGNPSSLTNTNTSNLFGGSKPQQTNNMMVNK
NLFSMGTTTSGLDGNKSIYDSMNSQSNLGNKNLFGASGVNNNSMQSGGGTVRNNLFMNTN
NQNNLNMKNLFDSGSNLNNAQGTNLSNKGLFGGLQQNNQSSSGGNLFGNLSSQTNPGGMY
SGLSPGANQNKGTGLFGTSGNTNQVSSTTSLFGGTSTMGQNKLGGVFGNLQSTDPNAQNS
GNTTNSLFGGMSGNQLKPMSAGSNIFGGMSTNTTTNTGTSNLFGNNNTASGMNQSKPGVF
FGNLQGSNQGTTGSSVFGNSSNNTSEMSQNKSGVFFGNLQGSNQGTTGNSMFGGMSSGMN
QNKPGGLLGNLQSPSQGTTGTTTNSSIFGSSVSGMNQNRGTTLFSGMSNTGASTTNTTNN
LFGGTSTMGQNKLGGAFGNLQSTDPNAQNSGNTTNSLFGGMPGNQLKPMSAGSNLFGGMS
TNTTTNTGTSNLFGNNNTASGMNQSKPSSNIFGSLSSTTQPTGATTSSSSTTGNIFGSTQ
TANQGMGSNVFGSSMGMNQNKFGNIFGGTSANQSSTMSSTFGSTGHGTSGISPTSALGSG
TNTSTGLGGGLGISSGGIGGTGTNTGMNISNLTNTTGGTTTGISTISGSSNLFGGLSSTS
NTNTMSLGDNKNLFGSKPGFSLGTATNISGQSSTNLGLGTNLVSGLLSPSLNTTTSSGLG
TNIGSSTTGSTNLLTGLNGGIGSNSTLGVGATTSPTIGISSSSSSNILGSNNNALNNTSL
SGNLLSNGSGMNQTGISFLGDKNLIGESRGNMQNNLLTVDGKDKSNDLLTNKLNINKESS
ENLIKTTKFDDDLVISKKKENIFNKAYDDDNDISNDEINLIKNQDGTFYNYINLIINDNI
NDIQKTTFSLLNDHDSLMWDNFKSNIFKNFVDYLVNFKQKKNQKVIKSNVLDLDQHEFQI
LIWLYNINSYKIDFIPFKSFYYLLLSDTNLNYSKMFISNSDKSILPRNFVEINDHYYMNY
KSYFLKSQINKHHDNYSTLDKDGIGNSSNGGVSGNSISIGMNNSVLDIDSIYKQILKDIL
NSLLNMNLSITKENIKKKEKEEYYENGDPNNKNSANCGSYIYENLLINYLFGTLQHLHDK
FYKLEISNYLSKDLNQIDEYFVDTKSNEIFSYCYDNFYKNEMDKENCTIHFFFYFVNIMF
RCGNYIGLIQIIKNDEFIKNLNIDSYLNDFIILLIRILLLIYNQNNEISIFENMKIDIDC
SDIFKKKIHMSNLIN**FFHSILYLCSNNIYAYDLLCILF**SDIFYKKGNMYTNREKKIEMKN
IFYYVQKKKRNSNYVDNNSDRNSSNEDNYDSGGDNNNIERGSNSINNNKGGKGKGSERKG
FFLDVFTNMLHKSKKSKDDEDDEPFETMEINEEIEKLNRENMNTSNTEMLGITNKYSYNF
EYCNIETSIWIELSLFLSKNFDLYGSKFQENFDILDTNLSYYNYDDDNNAFLNDTKHRRD
MINSKIEELFISISSCIINENREYFSVCSKLRADDVINNFIIVDGKISEKVKSNVFKVLR
TNF**LLYIKLFYYLLLVGNIYVTVGFL**SCISNNIQRILLVLTIFLHKNNVFENSKLNNIKI
KTLGFLKLKTDNNTILYNSKDINDNVPAGSDNMNLILLSMNNTDIPFDYFLLRNNNINIL
LKASYLLNLKTNICIKLLKSIVQENQGILLNESIIGHINNDGNIFYGKLHDFLFLFKNKV
KSRRNVKCFFLMHYVLYKKKIYNSNILRKIRKDIDNEYHYKCRNFKFVQNKNINTLNKIS
LDNSIINVHSSILYKISQFYSILAYFANQRKHYIVSFICYYILNDEQSAINSLLRIYNDE
LIYYYFNNEKEYGYIRKCAFRFYHLAKNMWPSNVKLESIEQKSYLILSILFMKKKMFEEA
FAIFSLALIPDNMLEKLSIADYYNIDLSSNFLVTLRELCKKNYKISELVDQSTIRSVIKF
LLPIKDKLDAQVAESINYLGSLSF

Figure S2. FG repeat *Plasmodium berghei* protein (sequence from release 28 of plasmodb.org). FG di-amino-acids are highlighted in blue. Two predicted transmembrane domains are displayed in red font. An internal repeat extending over the FG region is underlined.

***Plasmodium berghei* NUP138**
***P. falciparum* syntenic ortholog PF3D7_0903500**

>PBANKA_0417900 | Plasmodium berghei ANKA | conserved Plasmodium protein, unknown function | protein | length=1237
MNNNGGNANNPNMWGMPNNNLGNNSFFGTNMNSQNGLNENNNNSLNNQTNMTGNNMFFGA
SVNNQNNNIFENNMNSQNNVGKSDNSIFGTPSNDLNKSNNTSLFGSLSSNTSVNNNTTNA
FNANLFNAAKKDVYPGFTRSLLGNSPNNSSNIFKQSTLGSSIALGSQLNEDRGSESGGLF
SKEQLEAAKQIFANSSSGNLSSFNNKTNNNKLTFSGGFSSSSSAFSKNNINPFQSMAMQA
TNQNDKSSLMNNNMNKLFFGDTNNNNINGGGKINPFGMPTVNNLTSPNKLNEIGPGGFSQ
AIKSFNPNTNSLFSSQTNTGSTASTSFNNNDSSQKSLFSNFGGGFNSFAKTNTNDIFKKN
TTENATNNNNTNTSFSFLPSFNGSKNNDNSFSLNSFNATNNEKSSGSLFSFNNNNTNTAS
PDFSLFSNNNINKTTTPDNSINSSINKDAENKAIEKSNQNIGESKDSGITSVVSESFKAN
DASGTDKSIFSKETEAEKDKEKAGDKNEQNDNDKEKDETKETNVTLEIKKDENDSTKNHD
EKLIDHKSESKENQNNENEKDDKNDDKKYETSDNAKDNKDGTNNDKMSLFGKSLTFNSSF
FKTSTANLTDKKSNLESSSSLNNDTENKKEKTGFFFGNDDKNENTNTSTESKWDFSKFGN
NNLFNKEKESFSFSFKETSTTDKKNESTNLKDNKKEEDTKPSVSTESKDKNKEATATPKK
SIFNFGIKSNFLNQSSKDSKDDNINESNKEKDNEKPTKGAETDETDADGTKDHNTTGVYS
KLDSNDVGDKKKIWKLSFTKKKDQTSTDAQKELKENELKNNNLLNKGPSLQPSDITLGKG
SLFGSFTKDNEKKNEDTKNTKSDHFSNINSFGKNNTSISFNQSSLFGKPDEKGNTLFSSS
NNTFQFFNSSQNDKKKTEFQTKTQNIPEIKNNQNADDEDVNNVSNLINFISLEDRKKNVY
INNNAQCDDENHEKTYISSNLKSNNNNETSHRSQTANFQLKETKGTLRMDNNDDYADFND
IDFINGQQNLEINMEHQRQLENDRKSVENNIKTNECFLKKNLDQEMAVDVINNLSSFVKN
KINFMNYCSNEILDIYNKVSHYEKMYALISEDQIKIEKKQESLEKRLRLIQSEQCDMLSL
LNELDNENSLTFLKVLNQKNLNKDDSINNKNLYLDIDKFEKLADKIENLEELIDSIHNTS
KHDIVNDLVNKCYTNEINCEQIEKQLNGYSHELRNMK

Figure S3. FG repeat *Plasmodium berghei* protein (sequence from release 28 of plasmodb.org). FG di-amino-acids are highlighted in blue. An internal repeat at the C-terminus is underlined

***Plasmodium berghei* NUP205**
***P. falciparum* syntenic ortholog PF3D7_1364200**

>PBANKA_1140100 | Plasmodium berghei ANKA | conserved Plasmodium protein, unknown function | protein | length=1828
MDVEDNSMDFYSMSNYEDISNNKNTKLNDKEQGIISNKDRPISTNGITDLSCPLEKSCES
FVTVSDAYLSSSSLYKSNFYDDYEDNDAYNINNEVSTLRYSFSKNSSSDISNDSFSDDQY
ETPYINKKNNNINVKNCFNKNSHITYQDENNSNDNKICEGKSLNIENNLKNKISMLKKKT
QDISAFSKRENKTNESDDIYSNRGSNDDLDNSTHIENAKNGNKKTDVSENDSNMGGRKQN
NEHSDKSDQEDKNSKSENSDDDLYSVISGPENYDEIFLRTQKDDYYMNDNIKLKRNIFDE
NQNNSGHTKNIENEDDKNFDDTKTKYTTSTNFIKIIKFSDQLESIKKKNNDGNVFRSYDS
SQIEGGEDEKTIKEKKEKKKQKKNLNDKYNVQKDYEFKKLVRSTINMCHPKIKNIDENED
ILFNMDKISKIVIEDNNKDAYKTDDSENDDDCDTNNSLYDRVDDILDDRNKYIREKRDKY
FSEVKNGLNIGTNNNGDGIRIKNPSDKNGDHLNSVIGTIKNNDAINNCLKTENNKNNEEQ
YIKTNMANSVYYLNHDMIEINENVLKLYSGMCNYVERNNCTKNIVRIVPHLNNIYDNKKL
CYIKDSPSFSEIYKVIPEWKDSFELLNKKAQTDTEDMKNILKRIENIKNDLNILNKDLVD
DKKEFENIKFETIQHESMISNFEKKNKTYIKGVLKLDNMSFKLKKIHKINTNNLNNFNIT
ENVNRLLKYVDNAMTDYSQLFNEEKSIEILRFQQLDNTFNILTNTDDEYSFLMNLLKDEL
KEMICEKKEQLQNIIRRTMLYKAFDTTSFKDQITNTLNKQTLLINQIKYNLQCNVLLLFD
MYKKFSKKNIKIIQIPENYVKDYTNEVIKKSYIIKTFNDTKKNVSKYFNCVHFTRNRPIT
HPFFIHFCLGEKFLYHFINFVLYPLSEENQKKFMQYCSIPGFNSFLFVDKVLKLHKEEQQ
FPSTDLTYDFSSAYDRIGKTDYNDTSLINQYMQKWNELNPYLLALRNASEFCFQPSSNDI
NDNNDDYTSYTLMDSKKNVFDLINHIPDSIIGMSNAATAAAAIASSSGICNNINPSLGFS
SSFDNMNKLNSTFSLLNNQMSNNTNTMFNNITGVGTNISGLNSGNSFASNNNLFANNSGS
NLFGSTFNNSTNTTMRNNSTSYGTTNNNLLSGGNSSQSMFNTNFPKSSTSSIFGNASSTS
SFMGSGINNTPNVSVGGSTSLFGGSANLGNNMLNTNNRNPLENNSAVGIFGSSNYSTSAN
NNTLTVSTPFGTTNNDLLSGGNSSQSMFNTNFPKSFASSIFGNTSSTSNFMGSGINNTPN
VSVGGSTSIFGGSTNLGNNILNTNNRSNNILNIPNTPTMGNQQNSLLINNNATGNNNSGL
FNKSTLGSSSLFNSSMNNNANSQFNRTTLNTASSSLFANSITPNNNNILGSNVGMNNNTS
SIFSGLNNNKTAMLSNNNMFGNNLNNNTNSSGLNTGLFSLSSDTNKIGNNNNSNMSNSLF
TNSSGMSRINTNNNNNNNNSLFSNVNNYGASSNKNMVNTSSFNRGINMGSNNTISSYDLG
GNTNFGSFMGGNTSGNNSTFSGNNNYGGLFGGNNAQQGASSSIFNNNNTSMFSNNTRDTN
IFNNSNNSPSKNMFNNRMSYNSTNNITSNNSSTFGNKTGFSGSNNLNQFSTTLNNSSNTS
GNNSSNTSGNNSMFLSNNNMMRNKSSFPLSNNNYMSSSNLSNNNSLFQKNTFSSTQNNRD
SLSGFNTLNNNSSSNNFGNKFQQNVGNTFGSTFGNTSNNNMFSNNNSSSFGANTNTGLGA
NNNSVFQQTNSNFQYNFKPQNNSSLFSR

Figure S4. FG repeat *Plasmodium berghei* protein (sequence from release 28 of plasmodb.org). FG di-amino-acids are highlighted in blue. Two internal repeats—one at the N-terminus, one closer to the C-terminus—are underlined.

Plasmodium berghei NUP313
P. falciparum syntenic ortholog PF3D7_1446500

```
>PBANKA_1310200  | Plasmodium berghei ANKA | conserved Plasmodium protein, unknown
function | protein  | length=2715
MINNKRKRAYIQNERHENSNESESYVKLENEKGHDKDIIYDQIYSIPKKNKPRCLVLSNS
KDNLIANFTNLLKIDNYGVEFKTKWYVHLFRICLFSERLYGPTQTTGSGKNKNEKGNEEN
INKLILEYNVDELLKNVQKLILRKIDCENLYYQKILVIVCLLFDYFQNKKYHNIKTVKHL
EGEEEDTKNSIENFETKNGSDIHINDNSKKKIKSKKRYLETTKKQIYDDSGDDTTNKDKL
SNYSENNKLLGSELLKNNEKNKFKPIDIINNQKNYYIIEFKNILYNEKKKFYNLEIFYHI
TNLIIHKYWYSQEYAHYNIFIWFLNYINNKLEIKKSINNINSKLEMFINLHPKIFNISHA
KKKRKKNNYQHNNIIHNDINPTNPNNSHYFEGGIVDYPYIKNRFKQLGGGFSSFTNPINS
STPSNIINNNNNNNNNNNFFGVNNNSTNNTFNGFKSTNTDTSTIGNTFGSTFKGFGQSNN
ASIFGNNNTISNTVTKNIETGSTPFTSTNNTNTSLFGNSINSTSTTNNTNNSIPKFPSLF
PPNSSGSTNNNMNNINNNSFNSQLNKPLFANITPIGNNMMPNLQNKNVNLFGSINNNAPG
PNNSNINTTLDNNNNNNNNASIFGFDKNNQNNTSIFGNPSLNNTGTNTNFISASNNNATN
NIFSFNKDSQKKLFQTPQISNLENNNNNSSNNNNLLNTTTSNGNNTALFGQKASGTGFFN
STINGNINKDLLLNNKAGFENDMLLQKTNMFSNTKPADSINFMNNNNNMINTNNTNISSL
NNNAVSSFNFVTGNSNDILGGVSSNLNGQQNTNNFNNLFNTDNGSKELFKDLKITNEPTS
KFTVNKKPLFSRRPVKSNLLISNSITSENNIFSSTYSNKDSLLNNQSKPVKVIFGQNVNT
STPITNENTNTAIFGGSSNTTTNNPNNSTYTNTMNQIPSISTKEIKPITSTMDKGNIFNQ
ENNKNIFSNTNKLPFNASNNNTTMFGSTNCNIFGKSEISNIFSTSANNNTISISDTKTNP
DANANTNVNDNNLIGTNTSNSVFGNTSIFGQNEINNNDQNNFQGINFPVKKLTLEERKKK
TAKNNMLSLFSTNTNNSNIQTQNSITLNDTSLLINKNNNDTKKTNIFQFKSTQSDINTKT
DTSIFGGFNKSNIFDIGKSQENFEEKKFQFGNNKPSGNNTNIIRSIPNDTLFGSKNEDKQ
KTSDNTPKTTNLFTFENFTNKEKDESKNEVKNEVKNEIKNEVNDDKNQVKNEVNDDKNQV
KNEVNDDKNQVKNEVNDDKNQVKNEVNDDKNQVKNEVNDDKNQVKNEVNDDKNQVKNEAK
NENKIDITKEPTIFKGFGSILENQIDEANKSASKSFIFGLGIKEKDKEEKKTSLTSDINK
DDNALTTPKFGFTNNITTVSNNLNNIFNLGNNNKPDDNDNNNGNAKSSIFIERNEKEDGN
GAGLFASTTNNKQKDNANLFSFKAEKDTSNISGINRFSSTNLVGNGNKNKESDLTINYDN
IISRKRRKDVDLDYKDEIKNMNFSNLNENNDNNNLRNNETNHTNLNSQINNNMFLNEENK
NNEGLFLKKSKIGNTLNHTKHILNTIKFDNIPSKDKLSASFSENENNTNNDADQTDRNIF
NINMLISDQITKENELTNTITTSTLANNESNLNKSQLNFLSKNTGKNNNQYEADELDGKK
KTENQNNHHYQQNESNFCIPDGNKKSKLAEEKQNDFYEKMRINEYLMNNNYNSDENNKNN
NLEKKNTIVIDKKSMNRIKSYNKHISFDSIDVDLDRDVVDINELRQELFLCALNFHLHYW
AEKVILFYPHNEKINKYCTSIIKIKNKFDETYDFNLFDDIHSISRKLLKRLDKNSCIYES
VLFLSADNIQILKNAKLSLFDVFNIYHFWFKKNNSDLKKNFQNFLYTNKIYPSYLNYYMS
LYKNSKTSNNKFTDLKGNRTKIVKGLTPQNKLTDNEETQKHSSPALSFYEKFSQFSYSKQ
NSKSDLHIPNDSESDKNSTSENEHNNSVIDNEQSESYSTNDENTDNSENSTNSESQENSQ
IYRNFENSDLNDENRIKKKKKKITENNLAEIFNSDSSDASNVLENNYDDEPFNMNINEKY
IYLEKEEKRKKKLFLPSHIKIKKLSPCECILLELILKDNVLNVLKKYKLFKKEKYEYLYV
NLIAMLKHYNYFSTYKSEDINVENGNNNFANIIPSNSVKDKMDKNHRQYINNIDKGSNLK
RISNCLETNCTNIKNDKINILKKICDKNLLYYINLQLKNIAHLLNYKEIEIACTYLNHIK
NNLFVIFQIPILLFNVLYDKLIKCYNDFSIMDNYFNNSLLYKDFRYEYILKTKIAYMLLN
KFNEQDDIENCIKFAIYFDNNRVKAHKNNIYNDYIFHKNLINSYIKVHENIVPSEWIEAK
YAKNGELSDINDSEYLDYYSFFFKDISKYIKLSIIDKIINSNIFSLRKENYTICQSSEDR
ENTNKKYKSNNGTNSGNNSNFPNNNTNENNETSQNDIEKKKKKFACYFMYMVRRSIMDDV
VNIYSSKGEYFYKKLLATLVNNNFTKDYEKSHNKHFHMFIQVANINIFMKNVFFEYINFF
KKKELYNIEKIKNEDEIMDNLVNVVKVNGRNFFVVINTLAEIATKIEKNIVKDVNIHILI
NLRNIIIDTFYLFKYVFNNREFSLSLIENLYKNVLNFNDTINSFFSDDVNFYPKEQIKDL
YTHIRHKFTSQNVIN
```

Figure S5. FG repeat *Plasmodium berghei* protein (sequence from release 28 of plasmodb.org). FG di-amino-acids are highlighted in blue; two internal repeats positioned at the C-terminus are underlined.

Toxplasma gondii NUP145

```
>TGGT1_203780  | Toxoplasma gondii GT1 | hypothetical protein | protein  | length=1475
MAFPSPFFSGAVPSAAGGPERRPNTQRPIHRADPYAASLLGRLASKLASSVSSFFSSAPA
RKSPSVGVSTSSSHAVRLAPVRGSAPSPSLWGASELSADGEATGREVTVVRQPTSSRQSL
AEVLNPSLWPRDNSFHSSLALPRQGEPACLHPGSDPAAQSHDVLTLSTSGAVHYLQSSSP
PDMSDRAGTQWRRLDAVPASTSVSPNGDMQMGWQSQVADQTTFVPRAAPLYGDGVRGLQV
HFPAAGDPLSNLEASASRLTQQQWLLQQGATGPQPVMEGTVIPTPAHTGYPPQSIERPVT
VSSRAFSGYAIPGEPLAQPALRPTFNGHHTRVAPAASHSLFPTEERVASSLLAAGPIRRR
GSEARATPASGPPVPVLRLHPRRDSRSAAGSLLRQEEHLTVDQLQRLYLRCQPLRWRVRL
RELFRQEVESAGAVGVVGDALSGAPQDRATGHKSGQGSQVDTEGVVSVQDDLRRMDDSAG
RGTSSGPDTASGSASCDKSEKESAASNGPQGSASGVPEGMKAGEDRVTNQGSTEGTSPVK
GGLFGSLGQMDTKTSFATERRGGILETSKELPATGFGESVDAAGTTPQSSLFAGDRRLGD
SGAQSLFGGTAARAEDGLSAGGVTTNSKEIAREPPAPFSFPAVGGGAGGTAPLFSSGKAA
DQDTASQGRQDSQMGIKASGLGAASVSDQAYATLHASTLLGASGPAPGSIFQGSSSLSPP
ASTTMAGDKSEKQGSASGGSGLFGATKADSGGKGDTNASVSQSTAETPALSKPGAVSLFL
GGGSSSLTGGSNLFGKTPDQDAVSNDSQTLPTFGRPASSSLVSGSASETVTRGASLFGGA
TFSAVSQPPSTNSKLSSRQREPAEKPEESSSEEGITVSDGAEVGETASSSSNTASDRDAK
PQDRSAPGGQVTQSEASAVPWWQQNVGKACLVQVEDDGFAPDADDDADEKETGSAPGGGP
AASRHTFATPVSGGSLFSSSGTESKPATTPSSLFGTYSSMASTAASSSSSGAGGGLFGGV
TTSNLFGKSSSSTGILGTGTQSQTVSSSAPPSLFVFGGGGPAKTAGEAGKQTSGSALSVT
STASGPSGSLFGNSVSTAAPLAGGTAAAASSSAEKDKPSTVSLFGFKDSASATDSRVGGT
APAPKTSGTCLFVFGSTVGASQTACASSGGSVKRGREGCDIGAPASKSVFGGTTSQGAST
TGGLFGVSSAPSASLFGGNTSSGTGGGSSSSNLFVFGLSTNGANDAATKEAGPAALSSKG
ASPFGTQSSTPVFGGGTTATGSSSSLSSVFGASKADGNASNPFQFGKVPGGSILGAGTGS
LFGNGGTTSSTGTSLFGASLGAPGSGGSGSLFGAAAGGTATGGSESQRPGAAPVVSSPFG
SQSASGGGSSGGASVFGSAVASRPLTFGATGIGGGNQTSTGLGAGSAAGSTGTFGAATGA
GGLGQSGEDGNSLFGPQTGGPVIRRPRLTIKRTKK
```

Figure S6. FG repeat *Toxoplasma gondii* protein (sequence from release 29 of eupath.org). 29 FG di-amino-acids are highlighted in yellow.

Toxoplasma gondii NUP302

```
>TGGT1_259640  | Toxoplasma gondii GT1 | nucleoporin autopeptidase | protein  |
length=2894
MFSSNTSSSLTGGGSLFGSSGGGGGLFGGAGTQQQQGGSLFGGTGGFMSQPQQQTQGRLF
GNAGTTGMSLFGQQQTTQPQSGGLFGSSTTTNSGLFGSAPQQQTGGLFGSSGLSQPQQTP
SGGGLFGSTNTSTLGSSITGGGLFGQQPQQTGGLFGSSTGTGTGAAATGTGALGQQQSGS
LFGSSGFGTNTGGFGTSTLGTAGQQTSGGLFGSQPQGGGLFGSNTSSFGAAGTSGTSAFG
TSTFGGGAATGSVLGAGSLQQQQPFQPHKTEEGLLMSICFGNLAEVSQDEERWRFYQQRG
GGAMGASNTPGTGLFGQQTTQQPSGGLFGNSAGTTTSGGLFGSTPASTTGTGLLGSTPQT
NQQQSGGLFGTSTTGGFGGGLFGSSATTQPSGQQQTGGLFGNTGGSTTTPGGGLFGSSTL
GNTTGTGLFGSTTTPQQTQSGGLFGQQQQTAGSGGLFGSSSLTGATNTGGGLFGSSTTPS
TGLFGSTTQQCQPGATTTQTGGGLFGSTTTSATTTGQATTGTGLFGGLSSGTGATQGGGL
FGSGGTATTGATGGGLFGNAQQQSNLSGGGLFGSSGGLKLGTTTTPGTTPGATGTTGGLF
GNTTTTGASGGTSLFGSSLGTSGGGLFGSSAAKPGETGGLFGSSGTSGTSGSLFGNTTGT
AGTGTGLLGAAPASSGTGTSGGGLFGSSTTGSGTGLFGGSGIFGSAQTIQQQNSAAGGLF
GSTSAGGGVGGGSTLGSGQANQTSLFGAIGSLSGCGAQGAPASAAAADAYGLASLLGGHV
EVKLTLSARPSESSAQESAQIFPPRMQLLDPCGGTALGDTGLLGSVGSSVWRSGAPARLC
RGRRFRPLGPMEGGLSGSYLSSGASNASFALPEVYIQKAFGTSRTASPLEDESKGGQANA
LRLLPDGKAFSAAPWASTTLERSAVEALLMQRRRKPGRLTPDQVSPWSSTALQFMRSNPF
YREQLAEVQASKDTMENKDRDTLPPTSQSAPAAAGVKETTLASEEVSAPASVSCPAVPGV
LPASAAVPDEKRGWTANVEVSPSAKKLAGLGISPIETFSLATPAGSPGTSPNGSRTGPAE
ECTRWHGAPPASGSVAAGLESVAACLPAPEDLRPVLTRPDYETVPSIEVLTGMTEQKLSR
VQDFSITRRGYGSIRWPGYTDLRGINLDEAVKIEKLEVTVYGNEAPPCGVGLNKRAVITL
KNCKPRSVKYLDTLTIQRPEDEAYVQDKQCQYVTKVRRYTERMGAKFVDLNLATGEWTFE
VEHFSTYRFLDEDDENDEELETQLRQKALLPSTSAHQPTLESPVKVLPAHADSFPASPEN
RTRDFGEAPERITHLFPERPANGYSSERSSFLGVQKQVLKAGFAEADRETPAYLGGGVAE
NLVNPEFLKNVSLAESVNRHCQQRWRPLYVSGEAPSRGLKRVSYNSGKTVEAVHDLKEKA
LPGGERWEATGVSGGRMVWEPQVNGVEASHRGVQRFQDEKTEDKGVQDTERRDGGAAASL
RLEDGTGGSVRAVPPGRPCEDTRSSSASFPHLQQRRHCPYPVRCAPVISRDGLMALPLLS
LPVSLEGITTASLPGSLVQLAHLSPLLREEEMPVQNGGISSSVGSLEGGSAGLEAPVQPS
PMWTVGEVPDESVVSREEEEKQALSMAAQRYVQPLREAKGAPSLTAASLPLADVSPALVA
ASRLRPFQADLATSGAPYTNQSPTEQLSLVRDGCRVRRTLGASAMPLVLSAFLEEVVKEN
GESELQSREEDSQQSESSARADATQEFLPALTPAPEVAEMGKEGRRGRRSKGEAGSSGDS
NRRRFRSQQSEWWLLTELCRQESHALEKSRRSKSVKTLNAFPSSGLVQRLLLRLLAFFEQ
QTRVYSGCMSPTSSVSVGNSKCSLPLSSQPFLQSAPVSSAHPPDVPEASYLAPYMLQTWQ
LLVALMLSSPEQEESVFTAPSGLFSQRDISPEAILESQRQSRLLEWLLRESGREVTALLQ
RAAALRASPPFSTSEGLSLDRGESLCGATELCRQRGAAQNPAITRLGANALFRLAGTAGQ
RALQIAMAADHDEERKLLAVFHLAAAGQLYDAVELLLNSRPGEPYYPHLALCLAAHVQQQ
VGREFLYYNLFRATAPSFLTPPPGIARLYRLLTPCSTRASPSPGSASVKAKEGEKRKRSI
SPGVVEPAEASRCASPSKRHAGATDRNSGAKHESTHALSLHASWKSGETTEANSVSGMGL
EHFVSWRHQLTASLVFSSASPLEDPRVLSTGAVVSSGGNAEKKVDSVDEGEKAAACTQED
TVTQTKGEGSAADVDMFQRIRTELTPPPQAADAPRELRRALLQFEHRLRFRENGDASQSG
VEFAVPASPAPLYRQQDEDGETQKDRMHDGLQGATSNIFDLQYGLLRMHAGLAQPSLSIF
DPSSHTPYGLDFFFAWTAGVVTLLHRGSARARVRLLKQTESEGTEAAEAIDAEDGLEQEE
ARQLHRLTVAFAAELECLPGCWPWACAALLFTPFCGRALQGMRALIGRHAAEFTCTGVGP
RQSGKESQGLQAFPEKAAFEERRREILRVLLEEVGVAQWWLDEADGLYALSQRKFMQAAF
LFYWAYLRLRLPLVRGFLGSPVAAPLREELLNLWSPTGAMERHLLRHAGRALLQCLPGFL
LAVLLQQIQHVEKETRVSRMRRALEKCERLTGSVTSGTTRALSDELRAYERQERREEPTE
VCDEMGSMPSVLILSTEAKMRLLLQILEAIRVKLAGGLPDLSQDANGTDGSRKGVEKDIL
ASCVVGGECRSAKFPVDLARLACMEKALRWLLKKQMRKQKQHNRTEQRTSPGSARVPSSD
AKLSGATAKSSLEEDGDRVDADFSAALLREVEKQTKHVEAMSDANRGFFPTDISPDEPAF
WIALKTALLEERRE
```

Figure S7. FG repeat *Toxoplasma gondii* protein (sequence from release 29 of eupath.org). 45 FG di-amino-acids are highlighted in yellow. The autoproteolytically conserved HFS domain (part of the larger Nucleoporin2 domain) found also in yeast Nup145 or human Nup98 is highlighted in green. At the C-terminus of the protein (amino acids 2309-2571) lies a Nup96 or Nup145C domain.

Toxoplasma gondii NUP69

>TGGT1_273850 | Toxoplasma gondii GT1 | hypothetical protein | protein | length=723
MFGNTATTSSAASGGGGGGLFGSTSSPFASSSSSLFGGSSGTSKPGGLFGAAPAGSGTAT
PSLFGGTTGGASLFGAASTSSVSSGQTLSSPSTGSGLFGSASAAKPSLFGSTVPASTASA
QAASTGTASGSGSALFGALAKPAGADATGAPAGGLFGSSTGAQGTGGAAAATTAPAAGTG
LFGATSSASQPSTAGTGLFGAASSASQPSTAGTGLFGATSSASQPSTAGTGLFGAASSAS
QPSTAGTGLFGATSSSSQPSTAGTGLFGAASSSSQPSTAGTGLFGAASSASQPSTAGTGL
FGAAAGSSQPSTAGTGLFGAASSASQPSTAGTGLFGAASSASQPSTAGTGLFGAKPAADG
AGAHTAEKQATAKGTEAPAASAAPAEGTKATGGATGLLGSSSLFGDTKAASAASSTGATT
SLFGAAAAPAAGGATGTKPAGDSASSSSVSAPFGAGLGASSTANSGSSGNAAAGGLFGAA
AAGSAKAPEKLQGASGPAGATASG**ATTAETLPPPQQVALETLQHERVEEVLAKWEKRLQR**
RVRRFNEVAEEVGSVEKAMIEESKKLHALREEQIKIEKRQTYICDFIDGLERQQRDLLTL
LASVEASVLRQIPQDNGDPTGAAGGDALAQRVQREWEAESGFHSSSEEELLSRRLRNIDE
QLNDVGLALSEATERFQPGPLGTVAQVLGIHQAALQASWRQASELQQRMDALQRLTSDAK
HGE

Figure S8A. FG repeat *Toxoplasma gondii* protein (sequence from release 29 of eupath.org). 25 FG di-amino-acids are highlighted in yellow. An internal repeat region (see below) is underlined; at the C-terminus lies an NSP1_C domain (in bold) which can bindNup57 and Nup82 in yeast (https://www.ncbi.nlm.nih.gov/pubmed/11689687).

SSASQPSTAGTGLFGAA
SSASQPSTAGTGLFGAT
SSASQPSTAGTGLFGAA
SSASQPSTAGTGLFGAT
SSSSQPSTAGTGLFGAA
SSSSQPSTAGTGLFGAA
SSASQPSTAGTGLFGAA
AGSSQPSTAGTGLFGAA
SSASQPSTAGTGLFGAA
SSASQPSTAGTGLFGAK

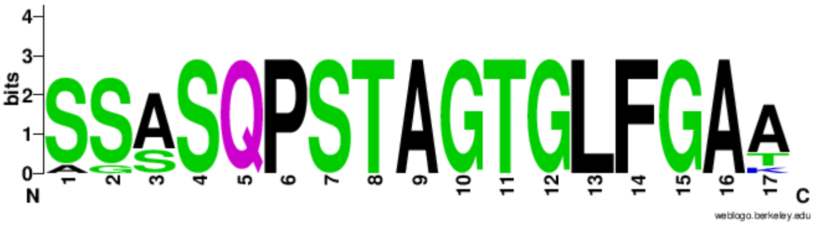

Figure S8B. The internal repeat region of TGGT1_273850 is composed of 10 near-perfect 17 amio acid repeats of the format SSASQPSTAGTGLFGAA; a weblogo of the repeats is shown next to it.

Toxoplasma gondii NUP206

```
>TGGT1_305790  | Toxoplasma gondii GT1 | hypothetical protein | protein  | length=1918
MASDKKDARSKSSKSEKVAKSKKSKSSRSGSGKSSSKDGGEKSAKSASKACGSTSEVKPC
EEQVSIPTQVGEPQTDTASLAPTNTPPPEQTFAVSVEPVTWEAQVPAAEEDVEASVNASA
EPVVAAEPDMPHPAASAAASADENPLVQTEQTPVAGERKTDANNLAPPPARQPDSMPSPT
RGMGPRGRLLAGSSSPRAAFGASQPPPPSFGVPPPPPVGGVALLDGQTQKKGEHSRRHLN
ARLMGDGTYETERVGRIQFVSMARTCIGLHDESGAPARSNCPPCSYLHDVVGMVCRRPPG
GTLPDRWVDYQDMHDELVLKQIREEWRRKEAEKADTVVNAEVAENVSIFPCKSFLARNFA
ETLPEGVRLLLPSRLQQRLNRRRRTLRHEKTGTEDLEGEISQDEEDVAAEGGRVCAKPRG
SPQGAAEPTEKSFADKHIKETPGADSRSATEGPETPEKAPAVEDNAQPASSEDAKAGAVE
TPADLAYEEDLTFLGNGGKVVAVINLCMTERYYSQPTLRRDGIEAYWIKVDGSGDIPDDK
VFCLFFRVIAFLAHKYSKLLPFSPESFATGWYSCGRDKMQEETVTVEDTPTGHAVCCDKF
TIVVHCTHGVNRTGLFVSLLLATLFNCSAEFAVKAYEAKRGAPLSKEVFVNWIRQKCKAG
IPERFRSALMPPEVSSLFTRVQKLLLEGGDLAGEEELTLPTPETLTNALDGERKEQNGRQ
ERLEAHVERMRQLRAALKAKKAEEKGEGTHEGQEAKERDDQQAEGEEKAEGEKAEESKDE
QAASAAEDSEEGQDAEETKENEELSRERALEVPKLPARLPADGIVLFGPIQSSLLAPEEL
LVSLLSSQESAKISGFEIMTGDDLHGQPHPYPQLLTTLRTRRRETPEKGRRGDGNKRRRG
AKGKAGAAEEETQGTGQQDGGPPSKEEMKGENGENEEACEEVEKKKEASVKSEKEAAAAS
DEDSKGHVSEDGTEHDTPAAKKRRLEAKLEDLEEAVKDEEDATAEMAEKKVEETQGEGTA
EQSAPAVPEPPNIDELSIPELHTKGQGLYSQKQIAYRLIQFREEDPLRLHYIVKVQCADI
ATFEWLLSHSFGRMRQQMIQGYPSSLYKLLYLRGKRRDKELFARAQEVAQAENAARQRNR
NVAAPKKGFRTGARPGAPGPFMGSRPGVAPPPPFPGIRPAGAVLPPPPAPFGPPVTPGFP
APPFLPPIPGMAPRGVPCAPGVSQPPYPPGASPPLFCPPPPGAPPAPGFGEGGRRDGPTP
PGPPGPFPGRLYEPRSPPPPGAEPPRMYDPDYVSKSAGEMGYRGYESQDRDFYGREPMKS
PQYPRGPGGETGGRRGPCGSWGPGGPEGDREGYHNQASSPTERGPYMRRGPGPAGFGGPE
GMRDGSLTRHGFGEEKRPFGPGCGGFGFEGTRGGRGPSDDGFGSGARGCNESGGRGPFPD
RSRRPPGDYEGRGRDDFDRGYGPQNRGGMYSNTGGTQGFEGPGFRGRGFGFSDESPGAGG
DRHEPGRVRGFSSDRFGDRVSSDRYGDRGDMWPRDGPRDQDEFRRFRDGSFRNAARDNKN
FSNFGYGNQNPAEFGGPMGERGGDFRARMESGSGVGGRGEDRGSRRISGWSSGFEDSRGN
SQFGRFSGGDRSGFDRFADSGERGSRFGDKEQAGGFGRRQGRPNVSRDFRRPPFGGSGSG
FADGSRDDGSRVPSLPGPPQWTIQKGPEAGGPSPGAFGAGHGQMGDAPGVEADKFRTGSA
PPGIGPTVESDSEDKSNLSASSVMQPQSFGIGGQESSAQNAFAQFAKQLASGLQDGGQGG
NSMAATMAMMGASGGSQQAMQQQLVALLSQQQMYGNSNLQGADMQQLMMYAQYYGCLLNG
MSEQQAAAVVQTQQLLQQQQQQLVNQQLLQQSIQQQQQLQLSLAGLMQTGTPKPDKKV
```

Figure S9. FG repeat *Toxoplasma gondii* protein (sequence from release 29 of eupath.org). 19 FG di-amino-acids are highlighted in yellow.

Toxoplasma gondii NUP67

```
>TGGT1_306560  | Toxoplasma gondii GT1 | hypothetical protein | protein  | length=698
MFSSATASGTSGGLFGSGTGGGLFGGSAATPAGASATAGTGLFGSAQATSGGLFGAASGG
AAGAAGTSRPGGGLFGGGTTTATGAGGTSLFGAAQQKPATGATTGLFGGGTAPAGTAGGG
GLFGNSQAAKPATGGLFGAQPAQSTGTTGGLFGAQSAQNTGAAGGLFGAQPAGATAASAG
AAVLPREPTVANIDRVVPGVLEKFKKIEEKMREEEKVMNELQAATRKMRESFSGFSSSLS
THQSRVSWALHHVLVLKREAQNLAPVVQRDKQLALQVEQLHQQLEQNVTAANASLARACS
AGGSRDAVYVVPLQVPCPLSTPLYQQLLQEVWSVSGKLQQLEQQVKLLRLERETAARSSC
SYTVDGVSLAGGYSESGVDFHSEVQMIKEVLESQREVLLATAQKALELRENTRRMQDYLE
RRGVKIPVFPEDQEEVAVNAMAVQATQTTGSLFGSTGPTSSPLGGIFGPLSSQTTPAAGG
LFGTVAGASATPATGTGGGLFGGSAATPAGASATAGTGLFGSAQATSGGLFGAASGGAAG
AAGTSRPGGGLFGGGTTTATGAGGTSLFGAAQQKPATGATTGLFGGGTAPAGTAGGGGLF
GNSQAAKPATGGLFGAQPAQSTGATGGLFGAQSAQNTGAAGGLFGAQPAQNTGATGGLFG
AQPAQNTGTAGGLFGGQPSGTSGSSLFGNTGAGLFGAK
```

Figure S10. FG repeat *Toxoplasma gondii* protein (sequence from release 29 of eupath.org). 29 FG di-amino-acids (11 at the N-terminus; 16 at he C-terminus of the protein) are highlighted in yellow.

Toxoplasma gondii NUP43

```
>TGGT1_310610  | Toxoplasma gondii GT1 | hypothetical protein | protein  | length=435
MAAWPSSSFISSSWGGGAGTGGAVSFASLAASSSFPGGTAPGGNPVGLGGMNPFSPGQAA
FGSGAGPSNTLSTTQPNAFSGQSSSVLGASLTASTSPFGASSSSLFSSASSSFGASTLFG
SASSSSSSSPFGSSTLFGTSSSSPFGVSSSSSTSSLFGAPSSSSSSPFGVSSSSSTSSLF
GAPSSSSSSPFGVSSSSSTSSLFGAPSSSSSSPFGVSSSSLFGSSSSPFGTAGGTAPAAA
LEGGQGAKSEGTKFIDAWKNLEAAAKGGIWPFSSFGVAEAECLFVGLDISPEEHRYLFYQ
RPQSEWRALGEQIVATQLQHLRQFHALVGEKAAADRLSFEAAPPSVEFLRLVLGGSLPQT
SFSVPQETLQTPSLGALGAPPAMNKAEINQEVSNLESLGTPGARRDETDARGAAFAAPQF
EPGKIPDVPPPRELC
```

Figure S11. FG repeat *Toxoplasma gondii* protein (sequence from release 29 of eupath.org). 15 FG di-amino-acids are highlighted in yellow.

Toxoplasma gondii NUP593

```
>TGGT1_313430  | Toxoplasma gondii GT1 | hypothetical protein | protein  | length=5639
MSLFGGGGGSSGTAPGSKSLFGATTSGTSSGLLGASSSSSSSLFGGGGSSLFGNSSSSIF
GAGSTAKPAQTPGLFGSGATSSSFSASPSSLFGSAGATEKPAASGGLFGSTGANSSSSLF
SGGGAASAATSGGGLFGSAASGSASSSSSSSSSSLFGGGGGAANPAASGGLFAGTGASSS
SSSSSSLFGSGSSAEKPTASGGLFASPGANASSSLFSGGSTATGTSGGGLFGNAASANSS
SSSSSSSSSSSSSSSSLLFGSGGATETPATSGGLFGSKGANSSSSLFSGAGTASTGTSKG
GLFGNATSASSSSTSSASASSSSSSSSSSSLFGSAGATEKPAASGGLFGSTGANSSSSLF
SGGGAASAATSGGGLFGSAASGSASSSSSSSSSSLFGGGGGAANPAASGGLFAGTGASSS
SSSSSSLFGSGSSAEKPTASGGLFGAPATASSLLPGAAASAETSGSSIFGGAASASSSSS
TSSASSTSSASSSSASSSASSSSLLFGGGEGTAGAVPAKPSSLPGSSSLFGSGQSTKAAE
APQSSLFSSSRLASASSAGASASSTSASAPAVASSMAALAAGSSPEIRKCLVSYMHLKSV
LISLSLSECPAQVEELFSSPQLLQRFASLLSPFDPSVDRALPQNISADLHAAISAVRKQF
AQRAARRRSASDATDAARDRLVPSEAMIYEALSVYLLRKFNKQQQLLSPRFSAPFLQEAV
DPDDPVTMREVEDILVDEQVDFFWCLAFIFRTAASVAPSSSRTQAPKQRTPATLDSSSDK
SLSGQKSENRDAPASGPKRDAAKTSSLEQACFAFACCLVREKNIEEGLFRTYDELSRRFD
GLERSGRKRSALTEDTVKREAARASRSPALQQVAEEPETVKALAATLRIQKALLHCFAAF
YRARNVLEACAKEKPVSAAATLHRLASMVTDDSFVGALAASSSEDRRLLHSSRLRLAGQE
VADASALLLVAAYPFALFRGALEGHQEAREAQETLAQLGAADHFAALHRGVMTPLLNAFK
QRLEREGDSWDLLALSSIDPAGEPSQSVDSGDEAESRGPGYPSAEYPDALYEQFIAHPSV
VPIVLFAHATALALLSSEDKDASSAGRSSKEDCSLSAAEVDAFLQPLLLCGSPSFAAALE
ALVVVRDGLRQAGSAAAAADPLLSMQREILLEFVHLLLLHLPLHHFQGFEPLVHAVSGLV
DSPGVAEDLWLRFSSRSGAFLFSPSVAPDSAGPGASTGKAAALARVEPSCSQCGCWGDFV
SSLQRETRLTQRRSSGSRLASPPRYGGLYVLVDLLRCVSFPFGLHLLLQLVSALVPRSPA
SRVVEKASEAEAEDAGDDEEVVVDATSLAAFLAFLTEPLPGILLPPLPDLFLPADAGDER
ALRGETAENALPRPGPSPLRRPYISVGPVPLELCLAVMLGFQEPLVDDKREVGGQGREAF
FDASAVSGGAEVWSLLPRGVSAEVQGLPVWRLTNAGATACATTAAALRQRFELAEGRSED
SRNPTASLLANDSRPPCLDGEGWWGVSTDGARAQPALRVLEPGRDNATSRCGRLLSGVSA
SWGPEQSDFFPAGESGGDASAFMLLHALRVRLGGRETECEETGRSGKGEEREHNVELPCL
SLLRTAWFVFDAGLQFLTQTGKPLPDAALKSFIAASALFARLAAWPPAMRLVAELVIAEE
VYNVGGPAAAFSVNAATAAASSNVREILALFPRKAKASKTKGDSACTGEDDKAAGADAGE
SEASGDSAAAVDAFLESNRSSAAVAPFALMPRLFALACLAAQQQQLRCILPEALTGLRCC
LTPCSLQNIFHVFRLLWPVSELGPSSSSSLSPARECRSLFSRFSSPDEGRLETPLASRLP
LVLLQPQTSELLTVDLASPPEAVHTYWQLLMLISVGSRSRSEASSLRRSLFRPFSSLASP
SSGAFFPPEFFSLECFCPEFFSAPFLSVSEGVWGDPTGAGDCGARGGTAGLLSPFSLRRL
LHLLGEAMEEEKSVGAYPVLKSTLLLLLNLLQLCPPAALTLSWQQHAALSAATVSESARE
LLLRERERRRHEREESDPSLEGEAEEHPMFRAFLAPFRDLARMQFGAPDADVCGDGGLLC
PPDLAREETANKGGSLEESYWVLFPTPASRPANRRAGLFRGDREVIAVEPEEEAGTESRE
RTAAETGADLTFLAECAESQRLRVFLERRQLTQLEEQRDTFGAICEFLFSSVWTRLASCS
FHHFAHRAEVVELLLAATTETLSLLQGANRLPGAGEETPSSVGPQGHRPEDSRTKKSRGD
RQRDRDGDRERWRQCEALRGSEVDDERIGDLQRDADAPGTIKATLAPDGGAWVFTHAGEA
RAPHSRFERERDNRLQILFWDKQGGVFAKRAATLAVYGHVELTNRLLEQLSSSNFLSSLA
SLLRCNILLELCPPYARCQALPTEGSPFLFAFVNSLLLSMASLDFHSLSSVAWITPLAAT
MLGTPPVQARRLRSSFASPLLLLPPRAPCCSNARGESTARGDSTAPGCRCCAFCSSASSF
SFSSFPSAVGSSSGNLFGGVQAAPTFLRETALGLLPRVVSLPAGPSETPYSPVSSLGRWS
AHWTGRALVENVPLCLSASGLSAVSTFFAFRAAPSSRLLDTVPALLPAEAALCVSAVTSP
NTDRSQNILCLVAQAFLHLFLATAVSPTGAALGACEAATSSLSPPGTSSACDASLLLPPQ
RSPDGESLGRRKRKSLDTQSTSCDSAPGLLLSRLQRQLWKLAFSPASTLPEDLGISSSPL
WPDVPDLLPSHPGDVLAALQEQETFLLLSQGAATVRVSPAARRPVWRRSSGTTARAPANF
VQSVFALLEASSRLKVVTASLLNLLTVASLGLQRARRGKSETDPRGADCFPAGLGCEAVA
PGRAHVSSSPFAVCLTFAPKEVQTVLEREDARGQQVEKASEKKEVEKAASRWRRKRLRFV
DLLFPGGVLSKGLRDLYAKEPMKEYRLLLLQLLLLLLSEPSDANCAPLALHHDLIFLGGG
GGFLDGEEKEEPLLWTAVDFAQQILLDVLDTAAASSLGDAEGEKSPGSRTQLRLASLGDL
KLLRLGLLLLEQLCFSAGAQAVHLQLLQQTVLGVRRLESRGRRLHTHRGAGVDDAFVALT
GGKGGGRVEEKSVGGWKLLLRVAHSLLATWQQVDWTRARLWLAATACTPGVPESEDSGRV
SEGHETLLPLASVETPALDSGGSAVFSRLISSAGFDVPLYASLGASTATSGVHTPAFSRR
PSGNLRASVSGRDSAKAKSAAGGDEAGSGAGSASRSSLFPRASGDGGDKGPVFSPGFGDE
EEEELGFVASLAAIELCGAFQALLRILVSQMHALPLAVRVQAKASEAGKPHKSLSISDLF
VDHAPSLSDYREFFSLFFFLSSDPTLLAAFFPAQRKTHAPHTPSLGLSPLPCTQRSLLRL
LLWLRSRSISPAYLHQAQPRPPPASFSLVPRPGGDAAACVVPAASSPSPVSHPTQTASMG
GRGGHCSRRPTAAASASLESFSDFWTRRRLLRAAVEEGVGGEACAFLESLAATSATSETF
LAAERGRARNAPGSAFSPLLSPSLVRACGVLSSPEQHGPGFLFDRRSLVLLATHVTLQAF
PLCRPGVVSETERERLPSEWRQCGEDERSVYLHPHERVENLRPRGRRRGGAGGASRETDP
GELRRGDSFRSKETGRRREGNSSVEASFSGCLLGEAWIAGAFACRVGVGESLADAQLSLL
AAFFQLLCVSRERGVFPLYYLPKKEGNSSVQNALEAGEEGGRSAEPEKSREASFASSPLS
DVQPPFQAAVFPVLSSLVEICEREKSLSSASCGLFFRSEFFRLLVALALQLVTCNWTTDV
RGAAAPSRLLLRASQASLHAAEKSLSSSAFASRRASLSAPPLSPLLRHPEELDDFSQRAS
LTPSRRGGEVVVSFALCPAVASGAEGPSMDTSRSRDRSRSPSPSAALSFGEQSYFLSRFS
AKIQPLADATGSAGRAAFLSPLWQDEALKGAAGESAHPPLAFLAKPLFAAGVRQPCIPSA
EILDNLERAAALAEASECDAEDANEVDTRASGQKDEDEEKEKKAASLRRRLRASELLFAV
QKGLLEVVREKVYQKTCGEGVEGFSERFMERKREDQKPRKQVHLPAFASSLSFFPNAPSG
ADVDAPGVHLAGPGTARRSGVFSPRYPPVSDLQLSALLLASAISSETRQEKTKRTASSGS
PRPRHTEHPGPDAGAGPLAREKALHAICRRIQQATRAAGHQLPEEADLLDLLAALYGFTV
FSTVWSFLSLSSGADGALPSLQSSRNSDAREETCPSPLVTFWAEALESQLAELLDQTETV
WGRSAGASPSVSESAPAFFSECFAFSDSSAVSEETRIATLLAKTPGVPCLVRVSALLRLS
```

```
LPRLFLNALVSLLDASVAAVHARRREGDRHEEKKPAEPGVATPRLLHPLAASRRASEDGL
ASWRRASRAEGRDASSLSTETDADPTLEPFDPLVACALQRALERLPLRPRRRAVSFFSHS
KPPGKAPDVFEDDEAASDALEGDPLSSPPAPLRAFLQLPLVRFFSPLAGSLSAQIQQHIA
TLNSLFPGVAPVVTRELWASQHLLLRQPVARCISLSPLDAFGDRAASAGSGPESQRDEDS
LFSPKTSLLLHDASFLVEEVALRTLGSYVAFFALLLQSGVTPRRPGGAAALFSRAAAAAA
PLLDAGDGEGFFVELHTCRNSRGARQGAGLLEAPPTSERERCTDSIDSIANSHSDACRSS
LFSLAKSVSASHPSLPGALLDADFFISLLTLAPFQSFLHPLRTPAFLPLPRSPQKDVAPT
VSWLPAYEALVTSSQDASCVGKKIRRSPSHLLFCRLLGLLALGLRQLRPSSGGCASPSGF
IPGDSGVECGTQLTTEAAGRDRERARTAGLIRSLQLLDGRMQFVLGPQGFAQTPQLATLE
EAALYAQLLTLLPPWRSMEIEQQQLVWRWFSLLRDFALSVVHGLLLSLVDGTCVLRPVSL
LECLACQPPTASSPRFSAFCASPDDPEGRGESRERRAEHRQRRGFWGDGGFAEASLGEDT
GESRRVIPSTFDQRGIYLMLQICNHTVSAFSKFGFLLPLSHTLPPPPPFFLPGCACCSSS
FARFSSASSRWCRRVRAVSPKSYCALITSLFSSPRDEETRLASPSRAGRQGREGNGRKDK
PEEEKRGAALVREREADKAEGPQGEEQRVLERRRHLLRDADVESEIPPPEREAPTPRGGK
CAKRNAAARERRDFLPTADEVAQTLAAFVEIARSLAKALEELAREDRTPPLAVLTAGDRR
FLPLSLFLRCEGPYMNRVSTLCSSPRPSQRQTSGEMSLLASGWARSPTPGSVSPSHSSVC
GAGRASSVVSMTAAGFLSEETPRELARRDVLKDTGLKDGGGLGRDGASVVSTRRGPSTEL
QGGCASFSIVSAGRQRAEQRRSLQRRRRSSPTVTACRVDRVTPRGCVASLLVSKENEQRC
RGRDEGLVAPSLLGIGEEDEELEVVCPDLLLQDALWELLRDTLEKAMAAGTQIVNAFVAQ
NILRPYADAAKQFLLLLIHLGAVSGHPSMSSLPSPSSFLPAGAAHFLRATETKGEETGKE
IFSGASSRNFYATLLEYLLKRYAVEEKIFEAAQGVGFPFLLDLRGEKRKAEGADSDVES
```

Figure S12. FG repeat *Toxoplasma gondii* protein (sequence from release 29 of eupath.org). FG di-amino-acids are highlighted in yellow.

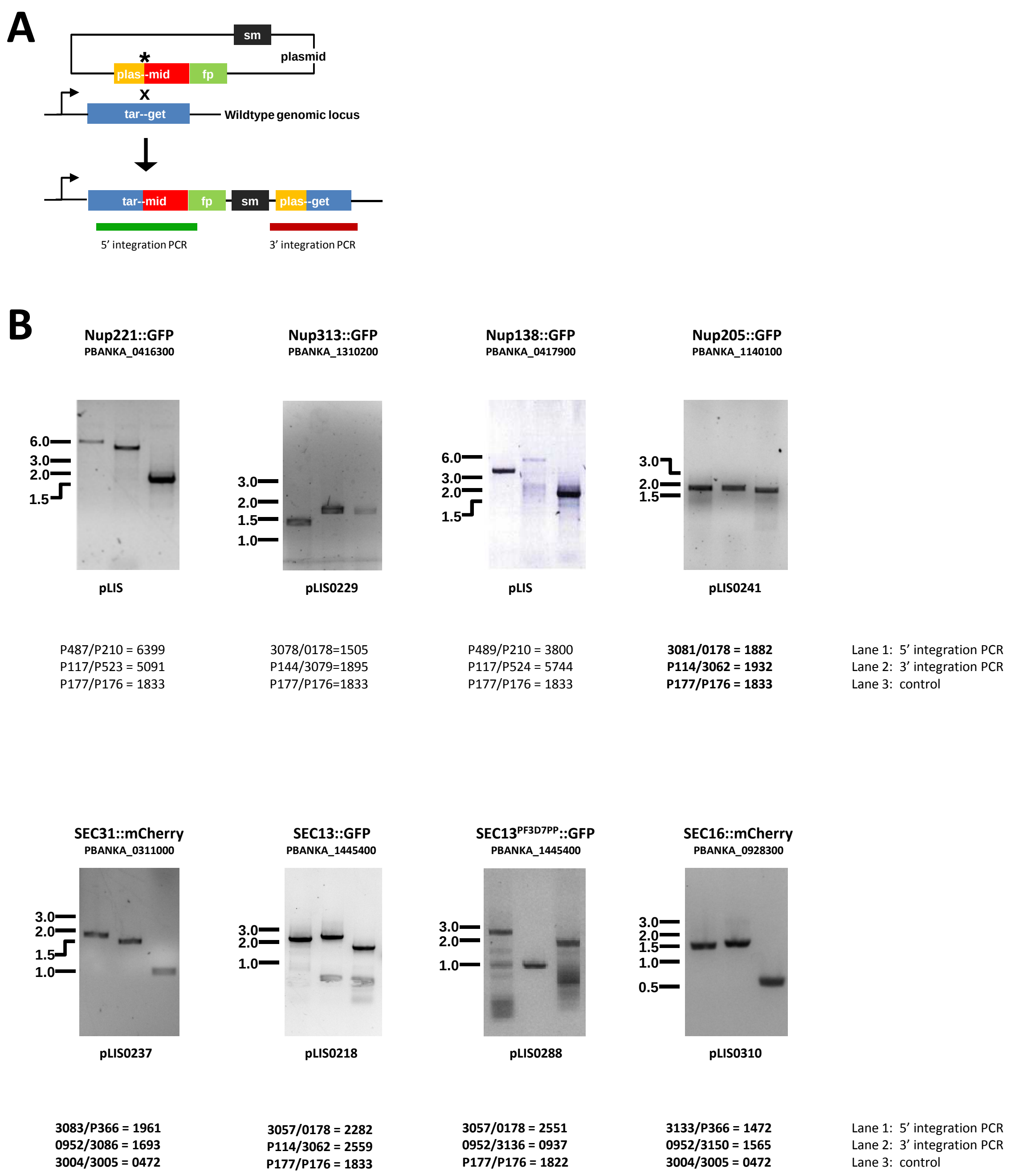

Figure S13. Polymerase Chain Reaction (PCR) genotyping of transgenic parasites. A Schematic overview of the *in situ* tagging strategy and indication of mutant-specific PCRs. **B** As shown for the top left panel: indicated are the tagged protein (Nup221::GFP) above the agarose gel separation of the PCR products with the size marker in kb; below the gel image are primer combinations and expected sizes of each of the three PCR reactions (5' integration, 3' integration and control)

```
PBANKA_031100    1 MALKSINISGNFDWCPFEEYKNYLICFNSHNLLYSNNGSLNNYTYLLDIN
PF3D7_0214100    1 MALKSINISGNFEWCPFEEYKNYLLCFNSHNLLYSNNNSLNNYIYLLDIN

PBANKA_031100   51 LNSDIRSLDIVHKLNFEEALSRE---------------NNKSNKTSSNEY
PF3D7_0214100   51 LNSEIRNLEIVNKYNFEDALKYDNDVIKGGNKKNNKNNKNNHNNNSVNEY

PBANKA_031100   86 VTSFEWINCNNFVESENENELNKGIIIGGLTNGNIILLNAQNLFDTNCVN
PF3D7_0214100  101 VTCFEWMNSNNFVDINNNEELSKGIIVGGLTNGDIVLLNAKNLFETNRN-

PBANKA_031100  136 YDNFILSQSNIHESSINCLECNKHKNNLIATGGNDGQLFIIDIENIFSPT
PF3D7_0214100  150 YDNFILSKTNIHDNGINCLEYNRHKNNLIATGGNDGQLFITDIENLYSPT

PBANKA_031100  186 SYDPYLDKNNLQKITCLNWNKKVSHILATSSNNGNTIIWDLKIKKSAVSF
PF3D7_0214100  200 SYDPYLDKNNLQKITCLNWNKKVSHILATSSNNGNTVIWDLKIKKSAVSF

PBANKA_031100  236 RDPHSRTKTSSLCWLENQPTQILISYDDDKNPCLQLWDLRNSNYPIKEII
PF3D7_0214100  250 RDPHSRTKTSSLSWLSNQPTQVLISYDDDKNPCLQLWDLRNSNYPIKEII

PBANKA_031100  286 GHSKGINNICFSSIDSNLLLSSGKDVTKCWYLNSNNFDIYNEVNNSGNNI
PF3D7_0214100  300 GHSKGINNICFSPIDTNLLLSSGKDVTKCWYLDNNNFDIFNEINNSANNI

PBANKA_031100  336 YSKWSPFIPDMFASSTNMDTIQINSINNGSKMTSKYIPTFYKKDASICFG
PF3D7_0214100  350 YSKWSPYIPDLFASSTNMDTIQINSINNGNKMTSKYIPTFYKKEAGICIG

PBANKA_031100  386 FGGKICLFDNIKSDNGSNLSASESASNKNANQLNQNIADEASMQINKNRG
PF3D7_0214100  400 FGGKICTFDNSTNNMSNVNNMNNVNNMNNINSFNNDNSCDGEYDSNKGK-

PBANKA_031100  436 LQNEGGNSNNPYLIKCHIYPTEVDLIEEADKFEKYIACGKYQEFCENKIA
PF3D7_0214100  449 ----NKSTQKKFLIKYHIYPTDMELISEADNFEKYITSGNYKEFCESKIN

PBANKA_031100  486 KCDDYHEKLTWKILQLLCTSQKEEIVKHIGYDMNEINQKIVESIGEESGF
PF3D7_0214100  495 KCDDDHEKLTWQILQLLCTSQRGDIVKYLGHDINNIVDKIMQTIGKQPGF

PBANKA_031100  536 IFKKYRCETNENMNNGGNITSNNLEIDSDMNNKHDNTRMNNLNNGENISG
PF3D7_0214100  545 IFKTLIDEKENNNNNNNNNSTNQMYQNDVLLHNDPNLMNNYLLKDNMNPN

PBANKA_031100  586 YSNYGNN-------------MHGEFVTSQMQENEPNFNEPFDLDPEKFFR
PF3D7_0214100  595 IMLNNNNNNINNRTGTNVMYSNGQNLLGDTNHNEENFNGNFDIDPEKFFR

PBANKA_031100  623 ELGEKTEIEKKQENETFKEDKNGKEDLKSSVNNVDSNTKDMISTIKCQNP
PF3D7_0214100  645 ELGEKTENEKIKQNEEDISGNDEHLLNSSIKGKENKTKNKKSGLGTDDNN

PBANKA_031100  673 EIGFGEINEDSKPN---------SNKTNSNNWNTGIESIIKECLLVGNIE
PF3D7_0214100  695 DNGDHNKNEGSNINGEHVSEHILNEKNNTNNWNLGIEAIIKECVLIGNIE

PBANKA_031100  714 AAVELCLYQNRMADALLLSSFGGENLWHKTKNIYIKKQNDSFLRNINYVL
PF3D7_0214100  745 TAVELCLHKNRMADALLLSSFGGEQLWHKTKTIYIKKQNDNFLKNINYVL

PBANKA_031100  764 DDKLEYLVKTIDLSSWDEALSILCTYAINNPNFNNLCEILAKRLQNEKFD
PF3D7_0214100  795 DDKLENLINNVDLNSWEEALSILCTYAINNPNFNSLCEMLAKRLQNEKFD

PBANKA_031100  814 VRSASICYLCASNFPETVEIWDSMPSSKSTLLNALQDIVEKITVLKMVIK
PF3D7_0214100  845 IRAASICYLCACNFSETVEIWNNMPSKKTSLLNVLQDIVEKMTILKMIIK

PBANKA_031100  864 YNKYNSTMNQKINQYAELLANSGRLKAAMTFLSFTENDNAIENLTLRDRI
PF3D7_0214100  895 YENFNSIMNQKISQYAELLANSGRLKAAMTFLCLIQHDQSIESLILRDRI

PBANKA_031100  914 FNSAAHIMPPHIKPPPSPFQYFDIKPFG---LSQKYNNSNNITNTSIHGI
```

```
PF3D7_0214100  945 YNSANHVLCQQIKPPISPFQIVDIKPSPNVYQNNMYNNNNNNNNNININSS

PBANKA_031100  961 YNKSQIHPNKSLTS-----------------------------------I
PF3D7_0214100  995 SNNNNNNNNNKVLSSMHHPMQQFNQCNVNKMYTSTSNIINNNTMNSNFKS

PBANKA_031100  976 VPPLPISEMHKQHGPSTPMVPFSHNSPTKFNTQMISGSSD-------ISR
PF3D7_0214100 1045 VIPPPLPMNTQMNNSTSSIQPPPSVPPTKFHTQIINNTMNSRSSIATTTK

PBANKA_031100 1019 LPPQKDFMGSINSTSNRGYPTSNFNNNVKYPSNGMGSLYIPPPNIPSHST
PF3D7_0214100 1095 NYPTSNLNSVIPTSMNNMNTNISHGNNVTPPYMSQTNVAVPNMNNNNNNN

PBANKA_031100 1069 TPINASSPPHTSVQNLAHLSSHSNLKLEHENKLPQGLNMFESQSYTNVNK
PF3D7_0214100 1145 NTMNPTYPSLPKFPNYNLNSQVQQNSIIPEKQLTSPMFSSNSYGNINKTH

PBANKA_031100 1119 AMQNSMAPPSNKIPNISRSSFSVSQNNIPPPFSQRNRHTSVSTSMGN---
PF3D7_0214100 1195 TTNNAVPPPPNVTSSVVTPPMPSNQLNNTRSSFADIQNVVSPPRNKNQSI

PBANKA_031100 1166 PIQQNIPFIQESQLNKRESIDSQVYNPVSPSQISPISQIQQTQQQNS---
PF3D7_0214100 1245 SSTANLNYQHDNQFNKRECMEQPVYPMTNQSSMFSMNNTMQKKNVPGGFQ

PBANKA_031100 1213 -FTPPQPYSQNKVGFTPPSGIKTTSPVAGAMSITPGMPVPWPIPTTTQQL
PF3D7_0214100 1295 DNTSQMNYGMQPTGSPPPSSLSTTSPIAGALTVTPGMPVPWPIPTTTQQL

PBANKA_031100 1262 GSTTQSTANENKKIQTVTKEQNGVLMNRSNIENVKRVISNLLNMYTSQEL
PF3D7_0214100 1345 GSTTQSTANENKKIQTATKEQNGVLMNRNHIENIKKTISNLLNIYTSQES

PBANKA_031100 1312 VKKKADDISVKVHELFDKIDNGAFNEQINTIIINLANSINENDFKTANKN
PF3D7_0214100 1395 VKKKADDVSSKVYELFEKLDCGAFNEQINDSLLNLVNCINANDFKTTNKI

PBANKA_031100 1362 LMEVSRNLWDGNNKAWIMGLKCIIPKC
PF3D7_0214100 1445 IVDLSRNLWDGSNKAWIMGVKHIIPKC
```

Polyproline-rich domain (bracketed: lines 976/1045 to 1213/1295)

Figure S14. ClustalW alignment of *P. berghei* and *P. falciparum* Sec31. Boxshading provided by http://www.ch.embnet.org/software/BOX_form.html. Note the high sequence conservation apart from a polyproline-rich domain close to the carboxyterminus

***Toxoplasma gondii* Sec13**

```
>TGGT1_201700  | Toxoplasma gondii GT1 | WD domain, G-beta repeat-containing protein |
protein  | length=654
MAAPATLSTFETSHAGCLHSVEFDFFATRLATASSDRTIRLWSLSTPEASTHAGEVAPKT
ATFLQELRGHEGPVWQVRWAHPSFGNLLASCGYDRRIIVWRQSAAAVPQGPQTRFAPTQS
LFTPVYTNEDHTASVNSIAFCPHEFGLHLAAGSSDGSVSVLSLSGDPGAPGAQAQLFWSR
KAFAAHFNGVNSVAWAPFMPAASQAGTPALMLATGGCDSQVRIWGLDPNSQEWQQLHQLT
DADPHTDWVRDVAFQPASASSLLLSSSRLLASCSEDGTVKLWVGEASTPSANPSATSYTW
SLLQTLRLHAPVWRVSWSVSGTILSVACGEKDVCLFRETVAGHWEKVSRLLGPDSLPQVA
PRPLPPAAAPGVPPTQPGQQTPQGPQLQTQAPQLQTQAPQLQTQAPQLQPQAPQLQPHGS
AAPLGAYPPSHPPSLSSSPPTHPAHGASHPPLSSFPSSHPSLPQNPAPGPLSATPPSTAA
TPRPLGPAAGQPPQGSPTPGVAFPAPGAPAYPGTPASAGLYGPPTPGAPGGAQSYPQPAF
AAPYPQGSAFPPAVQPAQTSLGGQQAPSPASFFPAGTAGSARPKPPAFYNIGAPEAGPTG
AAPPAAQPFFSGGQTPGAAPGLQPPTGFPRPPMAAPGAQAYAPRAPMYAPYKGN
```

Figure S15. Sec13 protein from *Toxoplasma gondii* (sequence from release 29 of eupath.org). 6 WD domains are present starting at amino acid position 3 up to 337 (underlined). The unusual C-terminal extension contains 81 proline residues highlighted in blue.

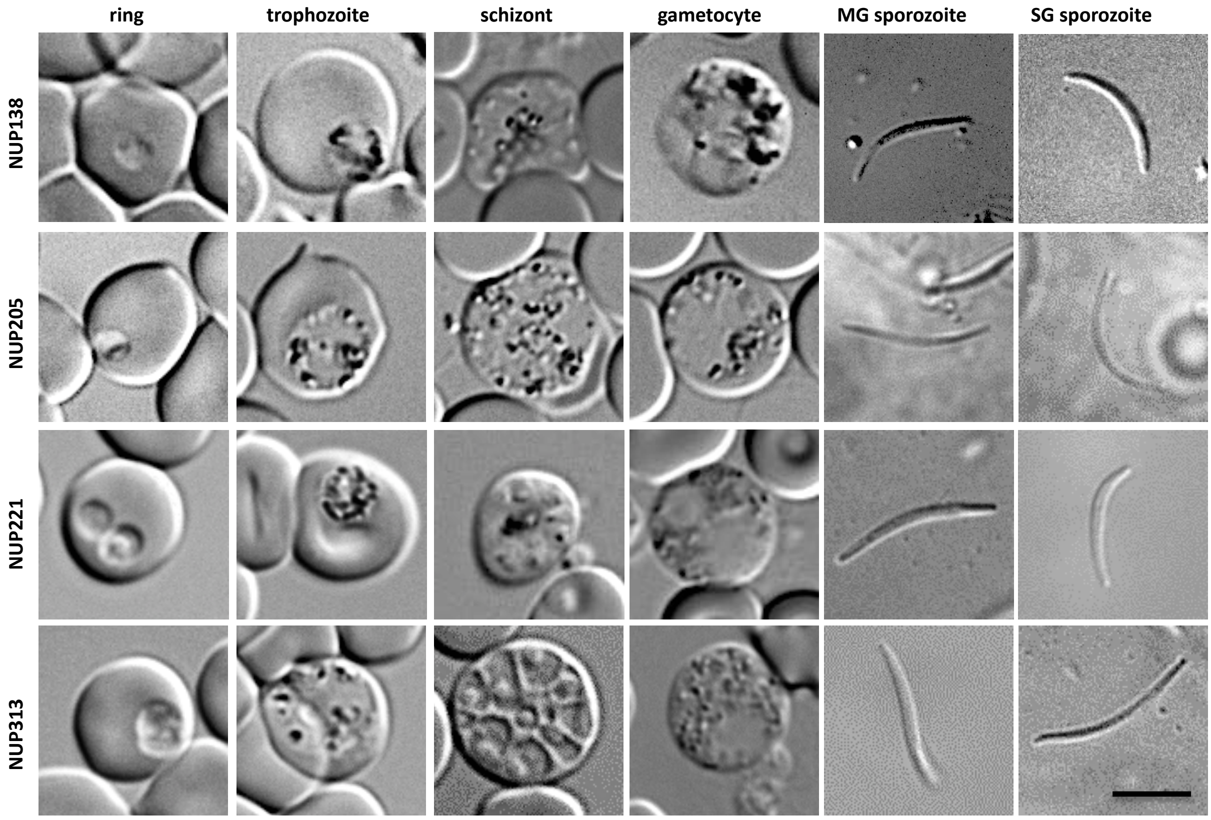

Figure S16: DIC Images to Figure 1B. Images taken with a Zeiss Axiovert microscope 63x. Scale bar 5µm.

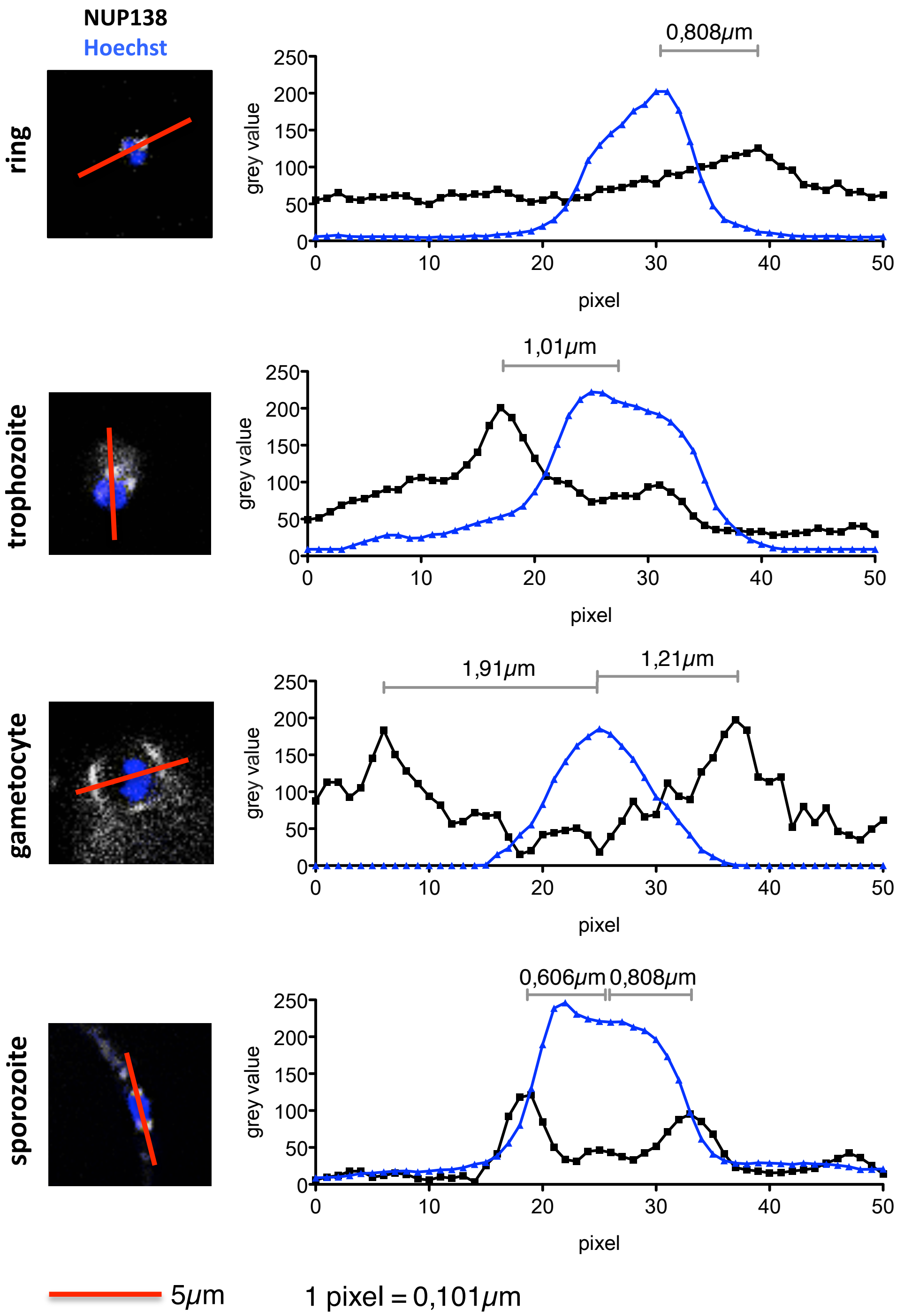

Figure S17: Position of the nuclear pore complex containing NUP138 relative to the DNA staining during life cycle progression as analyzed by line plots. Distances between signal peaks are indicated. Images taken with a Zeiss Axiovert Microscope 63x.
